# viralFlye: assembling viruses and identifying their hosts from long-read metagenomics data

**DOI:** 10.1186/s13059-021-02566-x

**Published:** 2022-02-21

**Authors:** Dmitry Antipov, Mikhail Rayko, Mikhail Kolmogorov, Pavel A. Pevzner

**Affiliations:** 1 Center for Algorithmic Biotechnology, Saint Petersburg State University, Saint Petersburg, Russia; 2 Department of Computer Science and Engineering, University of California at San Diego, La Jolla, USA

**Keywords:** Virus detection, Long reads, Assembly

## Abstract

Although the use of long-read sequencing improves the contiguity of assembled viral genomes compared to short-read methods, assembling complex viral communities remains an open problem. We describe the viralFlye tool for identification and analysis of metagenome-assembled viruses in long-read assemblies. We show it significantly improves viral assemblies and demonstrate that long-reads result in a much larger array of predicted virus-host associations as compared to short-read assemblies. We demonstrate that the identification of novel CRISPR arrays in bacterial genomes from a newly assembled metagenomic sample provides information for predicting novel hosts for novel viruses.

**Supplementary Information:**

The online version contains supplementary material available at (10.1186/s13059-021-02566-x).

## Background

Various metagenomic studies have greatly expanded the set of known viral genomes [1–6] and have raised the challenge of inferring the *metagenome-assembled viruses* (*MAVs*). Since the International Committee on Taxonomy of Viruses has proposed to include MAVs into viral taxonomy studies [7], there is a need for novel bioinformatics tools to accurately assemble, identify, verify, analyze, and classify MAVs.

So far, short-read sequencing has been the dominant technology for the discovery of novel MAVs [2]. Such discoveries are usually conducted by assembling a viral metagenome (virome) using general-purpose metagenomic assemblers (such as metaSPAdes [8] or Megahit [9]), or specialized viral assemblers (such as metaviralSPAdes [10]). Unfortunately, in the case of large, highly repetitive, or highly polymorphic viral genomes, it is often impossible to reconstruct complete viral genomes using short reads only [11]. For example, complete sequencing of giant viruses has been a challenging task [12, 13].

Modern short-read metagenomic assemblies rarely result in a complete assembly of even a single bacterial genome in a bacterial community. Recent progress in long-read sequencing technologies has resulted in many long-read metagenomic projects in the last two years [14–23] and led to the emergence of “complete metagenomics” [24, 25] that, in contrast to previous short-read metagenomics studies, aims to assemble dozens and even hundreds complete bacterial genomes in a bacterial community. However, there are still no specialized long-read assemblers aimed at sequencing viruses from metagenomes or metaviromes. As a result, recent studies aimed at assembling viral genomes from long-read metagenomic datasets [15, 20, 26, 27] used custom bioinformatics pipelines for viral sequencing that, as we show below, are far from being optimal. Another poorly addressed challenge is finding bacterial hosts for MAVs identified in long-read assemblies.

We modified metagenomic long-read assembler metaFlye [24] to improve the identification of MAVs in long-read metavirome/metagenome sequencing projects and developed additional tools for analyzing the identified viral contigs. We show that our viralFlye approach recovers up to 2.25 times more complete or nearly complete viral sequences, as compared to the previously published pipelines, while reducing the number of misassemblies. We also show that long reads improve the accuracy of virus-host association predictions (i.e., enable the host identification at a deeper taxonomic level) based on matching of the CRISPR-Cas sites. Interestingly, viralFlye also revealed many viral quasispecies, populations of multiple viral strains (species) that have structural variations or highly diverged regions. Although analysis of such populations may provide insights into how multi-strain viral communities are organized and how they evolve, viruses forming such populations typically remain below the radar of short-read studies.

## Results

### viralFlye pipeline

A complete viral genome can be represented as an isolated contig (either linear or circular). However, if a viral genome is heterogeneous (represented by multiple closely-related strains or species), it may form a *multi-edge connected component* in the metagenome *assembly graph*. In contrast to previous approaches that mainly focused on single contigs, the viralFlye pipeline analyzes both single viral contigs and multi-edge viral components. It includes the following steps described in the “Methods” section and illustrated in Fig. 1:

1. Launching the modified metaFlye assembler (that we refer to as metaFlye_v) aimed at viral genome sequencing.

**Fig. 1:**
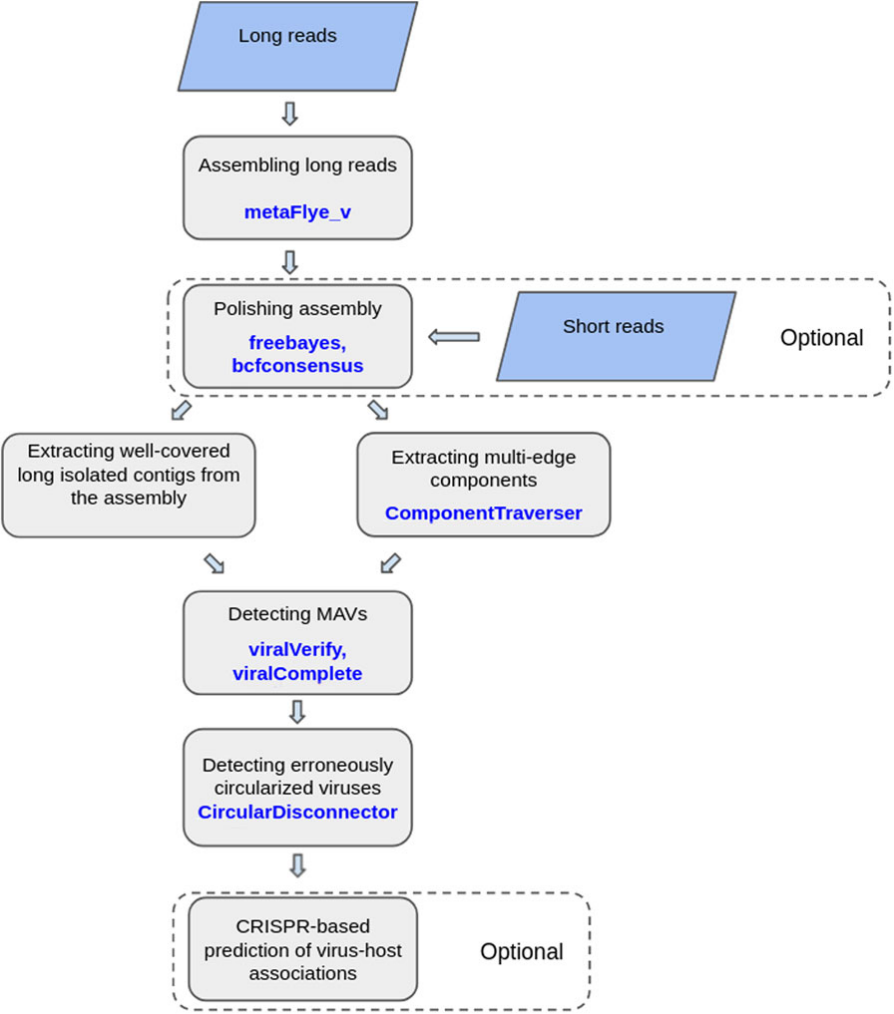
Graphical representation of the viralFlye pipeline. viralFlye takes a set of long reads (ONT, CLC PacBio, or HiFi Pacbio) as an input and assembles them using metaFlye_v. If short reads are available, they are used for polishing the metaFlye assembly with freebayes [36] and bcfconsensus [38]. Afterward, viralFlye extracts short (length 5kb–1Mb) and high-coverage (coverage > 10*x*) isolated contigs as well as small multi-edge connected components. It further analyzes them and identifies putative MAVs using viralVerify and viralComplete and classifies the inferred MAVs into linear and circular using the CircularDisconnector module. Finally, it performs a CRISPR-based prediction of the virus-host associations (optional step)
2. Polishing the metaFlye assembly with short reads (optional step if short reads are available).
3. Identifying isolated linear and circular contigs in the assembly graph.
4. Identifying multi-edge connected components in the assembly graph (the ComponentTraverser module).
5. Detecting MAVs by verifying the identified linear and circular contigs and connected components as MAVs using the viralVerify tool [10] and checking them for completeness using the viralComplete tool [10].
6. Classifying viral genomes into linear and circular (the CircularDisconnector module).
7. Predicting the virus-host associations based on CRISPR analysis (optional step).

### Datasets

We benchmarked viralFlye using the following four datasets that cover various types of the currently available long read technologies. All datasets were downloaded from SRA in the fastq format.

HUMAN_GUT: Long error-prone reads from fecal samples from twelve individuals analyzed in [15] (SRP098614). The HUMAN_GUT dataset contains ∼11 Gb of long reads per sample with an average subread length of 8 kb sequenced using the PacBio RS II (CLR) system, and 33.8M single short reads (average read length 219 bp) sequenced on Illumina MiSeq.

WARWICK_VIROME: Long error-prone reads from three marine water samples from English Channel, filtered using 0.22 mkm pore-size membrane, analyzed in [6]. DNA samples underwent whole genome amplification and were treated by S1 nuclease digestion to de-branch chimeric DNA formed during the rolling circle amplification process. MinION libraries were prepared using EXP-NBD104 and SQK-LSK109 kits and sequenced using MinION with a FLO-MIN106 flowcell. Datasets ERR6018294, ERR6018309, and ERR6018338 (referred to as WARWICK_VIROME1, WARWICK_VIROME2 and WARWICK_VIROME3) contain 5.8, 6, and 7.3 Gb of reads respectively

ALOHA_VIROME: Long error-prone reads from marine virome samples analyzed in [27]. Size-selected particles (30-kDa filter) were sequenced on the Oxford Nanopore GridION X5 with FLO-MIN106 (R 9.4.1) flowcells. As in [27], we analyzed the samples from the Station ALOHA (SRR10378147, SRR8811961, SRR8811964). These three ALOHA_VIROME datasets (referred to as ALOHA_VIROME1, ALOHA_VIROME2, and ALOHA_VIROME3) contain approximately 701, 341, and 558 thousands long reads, respectively (mean read length 17 kb). These long reads are complemented by 29, 28, and 24 millions of short Illumina reads, respectively (150 bp long reads with insert size 350 bp) that were used for polishing.

SHEEP_GUT: Long accurate PacBio HiFi reads from the sheep fecal sample analyzed in [24] (SRX7628648). The SHEEP_GUT dataset contains 3.6 millions reads with mean read length 13 kb, sequenced on a Pacific Biosciences Sequel instrument using v.2.1 chemistry (libraries in the 9–10 kbp range) or v.3.0 chemistry (libraries in the 12–16 kbp range) and 20-h movies (8-h pre-extension). These long reads are complemented by 512 millions of paired short reads (150 bp long reads with insert size 350 bp) generated on Illumina NextSeq 500.

### Benchmarking

We have compared viralFlye with Raven [28] and the earlier version of metaFlye, i.e., the version metaFlye 2.8.3 that does not include additional modifications for viral assembly implemented in metaFlye_v. Since Raven and metaFlye are general purpose assemblers that do not include a specialized virus identification module, we used the pipelines (referred as Raven+viralTools and metaFlye+viralTools) that combine Raven and metaFlye with the same downstream viral analysis steps as in the viralFlye pipeline. We evaluated the assemblies from all three pipeline configurations in terms of the number of reconstructed viral genomes and additionally used the checkV tool [29] as an orthogonal validation approach. As we show below, viralFlye recovered the largest number of viral genomes for nearly all analyzed datasets, and had the lowest rate of chimeric misassemblies.

We classify a contig as *high-coverage* if its coverage by reads exceeds the *minCoverage* threshold (default value 10x), and *low-coverage*, otherwise. Since the single-base accuracy of low-coverage contigs in assemblies of long error-prone is low, we focus on analyzing high-coverage contigs. We note that metaFlye outputs information about the contig coverage but Raven does not—it only provides the read count *readCount* for each contig. We thus estimate the contig coverage in the Raven assembly using the average read length for each dataset (computed as *readCount*×*readLength*/*contigLength*). This approach may result in a biased estimate of the coverage since the read length for all analyzed datasets has a rather high variance.

viralFlye extracts high-coverage circular and linear isolated contigs from the GFA assembly files with the lengths varying between *minLength* (default value 5 kb) and *maxLength* (default value 1 Mb). A user can change these default values, e.g., to analyze short circular ssDNA viruses, it would be useful to decrease the *minLength* parameter to 1.5 kb.

### Analyzing the HUMAN_GUT dataset

Table 1 presents information about the MAVs identified in the assembly graphs of all 12 samples in the HUMAN_GUT dataset. In total, viralFlye assembled 44 linear and 18 circular contigs as complete (i.e., their lengths are similar to the lengths of the closest viruses in the database). In comparison, only 12 viruses were assembled in the original study of the HUMAN_GUT dataset [15]. Below, we compare these assemblies with the viralFlye assemblies. Since metaFlye 2.8.3 produced empty output for 8 out of 12 samples (because it is not designed to assemble relatively short contigs), we provide its results only for the remaining 4 samples.

**Table 1:** Metagenome-assembled viruses in the HUMAN_GUT dataset

Sample	metaFlye +			viralFlye			Raven +			Viruses assembled in [15]
	viralTools						viralTools			
	Linear	Circular	Components	Linear	Circular	Components	Linear	Circular	Components	
apr34	0	7	0	2	6	0	4	3	0	4
apr38	0	1	0	0	2	0	2	0	0	0
MO1-2	-	-	-	1	0	0	2	0	1	0
GM1-2	-	-	-	5	0	1	1	0	0	0
FAKO27	-	-	-	11	2	0	5	2	0	2
YS1-2	0	3	0	1	3	0	3	1	0	2
ES_all	-	-	-	7	0	1	6	1	0	1
FAK02	0	1	0	2	3	0	1	1	0	0
FAK03	-	-	-	1	0	0	0	0	0	0
FAK05	-	-	-	3	0	1	1	0	0	1
GF1-2	-	-	-	5	0	3	4	1	0	1
FA1-2	-	-	-	6	2	1	2	1	0	1
Total	0	12	0	44	18	7	31	10	1	12
Contigs with “high” or “medium” completeness by checkV	0	12	0	35	18	6	29	10	1	12

The number of complete linear / circular viruses and complete viral multi-edge connected components are assessed using viralComplete. checkV is used for orthogonal completeness validation. metaFlye did not generate assemblies for 8 out of 12 samples

**Fig. 2:**
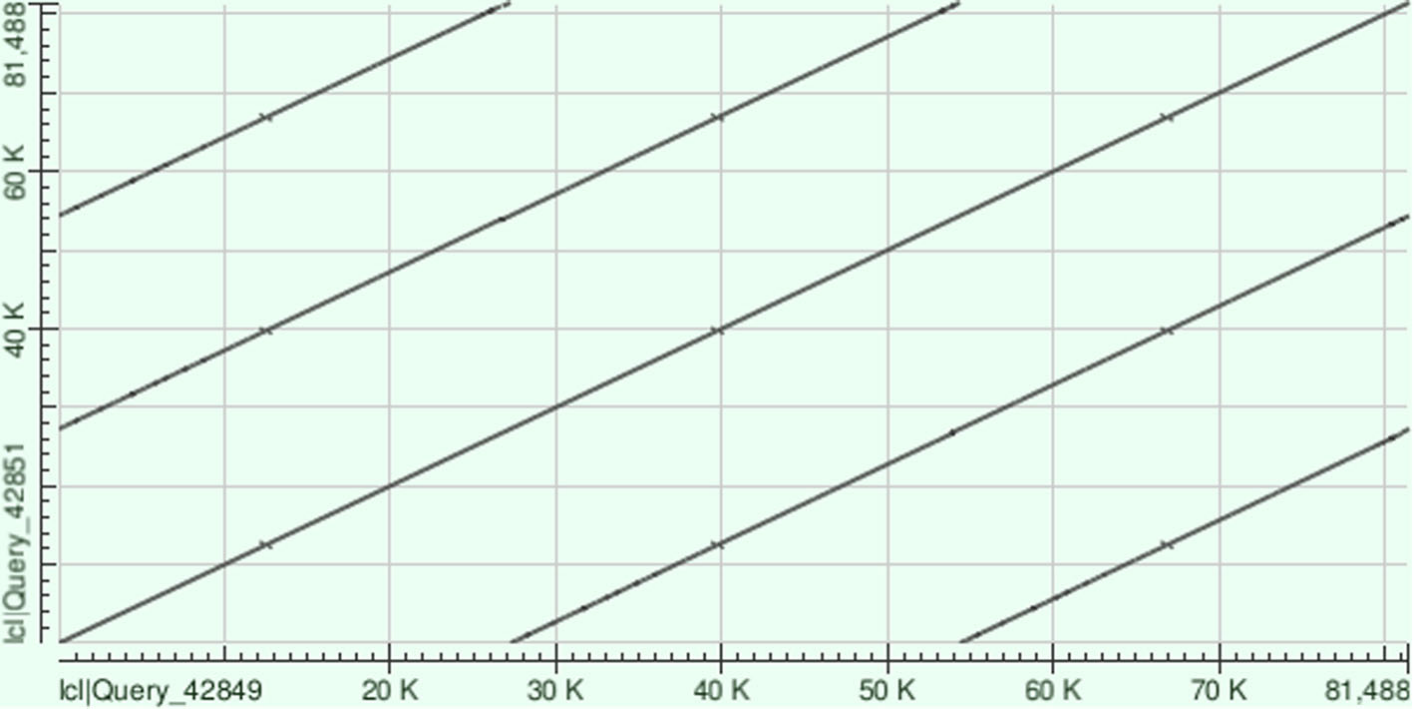
The dot plot of the alignment of MK415401.1 against itself reveals a triplicate repeat of length 27 kbp generated with BLAST. This 27 kbp sequence likely represents a complete viral genome (93% completeness according to checkV)

Nine viral genomes assembled in [15] (MK415399.1, MK415400.1, MK415402.1, MK415403.1, MK415404.1, MK415406.1, MK415407.1, MK415408.1, MK415410.1) were assembled into circular contigs by metaFlye_v. These 9 viruses include all 5 crAssphage-family viruses identified in [15]. Two viral genomes identified in [15] (MK415401.1 and MK415405.1) are likely misassembled. For example, alignment of MK415401.1 to itself (Fig. 2) reveals that this genome represents a nearly perfect triple repeat, suggesting that the real genome is three times shorter than MK415401.1. This shorter genome was assembled by metaFlye_v. The remaining viral genome (MK415409.1), that was assembled into a circular genome by metaFlye 2.8.3, is a part of a multi-edge connected component in the metaFlye_v assembly. Since metaFlye_v can recover less abundant viral strains, it is possible that the heterogeneity of this virus was more adequately reflected in the metaFlye_v assembly but prevented generating a single circular contig for this virus.

We launched CircularDisconnector on all 18 circular viral contigs assembled in the HUMAN_GUT dataset. For 4 out of 5 viruses from the crAssphage family, it identified *direct terminal repeats* (*DTRs* of length varying from 1905 bp to 2170 bp). Since DTRs often trigger misrepresentations of linear genomes by circular contigs, CircularDisconnector detects and linearizes the misrepresented circular contigs (see the “Methods” section). One more crAssphage virus (MK415408.1) has multiple coverage drops and jumps and thus was not classified as linear. No other incorrectly circularized viruses were detected.

### Analyzing the WARWICK_VIROME dataset

Table 2 presents the benchmarking results on the WARWICK_VIROME datasets. In the original publication [6], these datasets were assembled using an older version of the Flye assembler (v.2.6) that may generate inferior results as compared to metaFlye 2.8.3 and metaFlye_v. However, the original study does not report the per-sample statistics of the reconstructed viral contigs and only provides the total number of viral contigs reconstructed in both long-read and short-read assemblies. It is thus difficult to infer the number of contigs inferred from long-read assemblies only and to adequately compare with the viralFlye results, particularly since [6] reports many low-coverage viral genomes while viralFlye only reports genomes with coverage at least 10x reasonable single-base accuracy. Therefore, the larger total number of viral genomes reported in [6] (as compared to the number of viral genomes identified by viralFlye) likely reflects their reporting of viruses identified in the Illumina-only assemblies and the absence of the coverage cutoff.

**Table 2:** Metagenome-assembled viruses in the WARWICK_VIROME dataset identified in various assemblies

Sample	metaFlye +			viralFlye			Raven +		
	viralTools						viralTools		
	Linear	Circular	Components	Linear	Circular	Components	Linear	Circular	Components
WARWICK_VIROME1	15	23	111	16	26	137	0	0	0
WARWICK_VIROME2	60	20	124	37	32	169	0	0	0
WARWICK_VIROME3	38	22	108	34	27	150	0	0	0
Total	113	65	343	87	85	456	0	0	0
Contigs with “high” or ‘mMedium” completeness by checkV	89	58	238	58	76	282	0	0	0

Since the average read length in this dataset is low (≈ 2 kb), the coverage estimates for the Raven-assembled contigs are too low to pass the coverage cutoff 10x

Interestingly, the metaFlye pipeline identified more linear viruses but fewer viral connected components than viralFlye. We attribute this increase/decrease to a more adequate representation of low-coverage strains in the viralFlye pipeline that turns some contigs into connected components in the assembly graph.

### Analyzing the ALOHA_VIROME dataset

We used short reads in these datasets for polishing the long-read metaFlye_v assemblies (an optional step in viralFlye). In [27], the authors also utilized short reads for polishing but used an assembly-free approach for MAV generation that only considers viruses with DTRs covered by a single read.

Table 3 provides information about the reconstructed MAVs and compares the number of MAVs identified in our study and in the original study [27]. We further dereplicated the identified MAVs (to ensure that they indeed represent different viruses) using CD-HIT-EST v4.8.1 [30] with the similarity threshold of 0.95. No MAVs identified by viralFlye had similarity exceeding 0.95, implying that they represent different viruses. Some MAVs identified in [27] represent the same viruses, reducing the number of identified viruses in their study from 566 to 540 for ALOHA_VIROME1, from 93 to 92 for ALOHA_VIROME2, and from 1205 to 1114 for ALOHA_VIROME3.

**Table 3:** Metagenome-assembled viruses in the ALOHA_VIROME dataset identified in various assemblies

Sample	metaFlye +			viralFlye			Raven +			Viruses assembled in [27]
	viralTools						viralTools			
	Linear	Circular	Components	Linear	Circular	Components	Linear	Circular	Components	
ALOHA_VIROME1	368	306	75	1363	315	239	109	40	33	1205 (1114 unique)
ALOHA_VIROME2	259	125	32	876	96	127	17	10	10	566 (540 unique)
ALOHA_VIROME3	408	316	69	1577	280	196	91	44	17	93 (92 unique)
Total	1035	747	176	3816	691	562	217	94	60	1864
Contigs with “high” or “medium” completeness by checkV	964	719	148	3551	663	525	217	94	58	1837

We further used the assemblies from the original manuscript [27] to estimate the rate of chimeric misassemblies. We aligned the query contigs (in metaFlye, metaFlye_v, and Raven assemblies) against the original (reference) contigs. Since all reference contigs contain direct terminal repeats (DTRs), they likely represent complete viruses. A query contig that is collinear with a concatenation of two different reference contigs is thus classified as potentially chimeric. The metaFlye pipeline identified 81 potentially chimeric contigs, while viralFlye identified only 6, suggesting that the specialized viral assembler is less prone to misassemblies. The Raven pipeline identified 4 potentially chimeric contigs, but assembled an order of magnitude fewer viral genomes.

CircularDisconnector labeled 28, 4 and 26 circular contigs in ALOHA_VIROME1, ALOHA_VIROME2, and ALOHA_VIROME3 datasets as falsely circularized linear viral contigs. We hypothesize that there are many more linear contigs that were incorrectly classified as circular ones but evaded detection by CircularDisconnector due to highly uneven coverage.

### Analyzing the SHEEP_GUT dataset

**Fig. 3:**
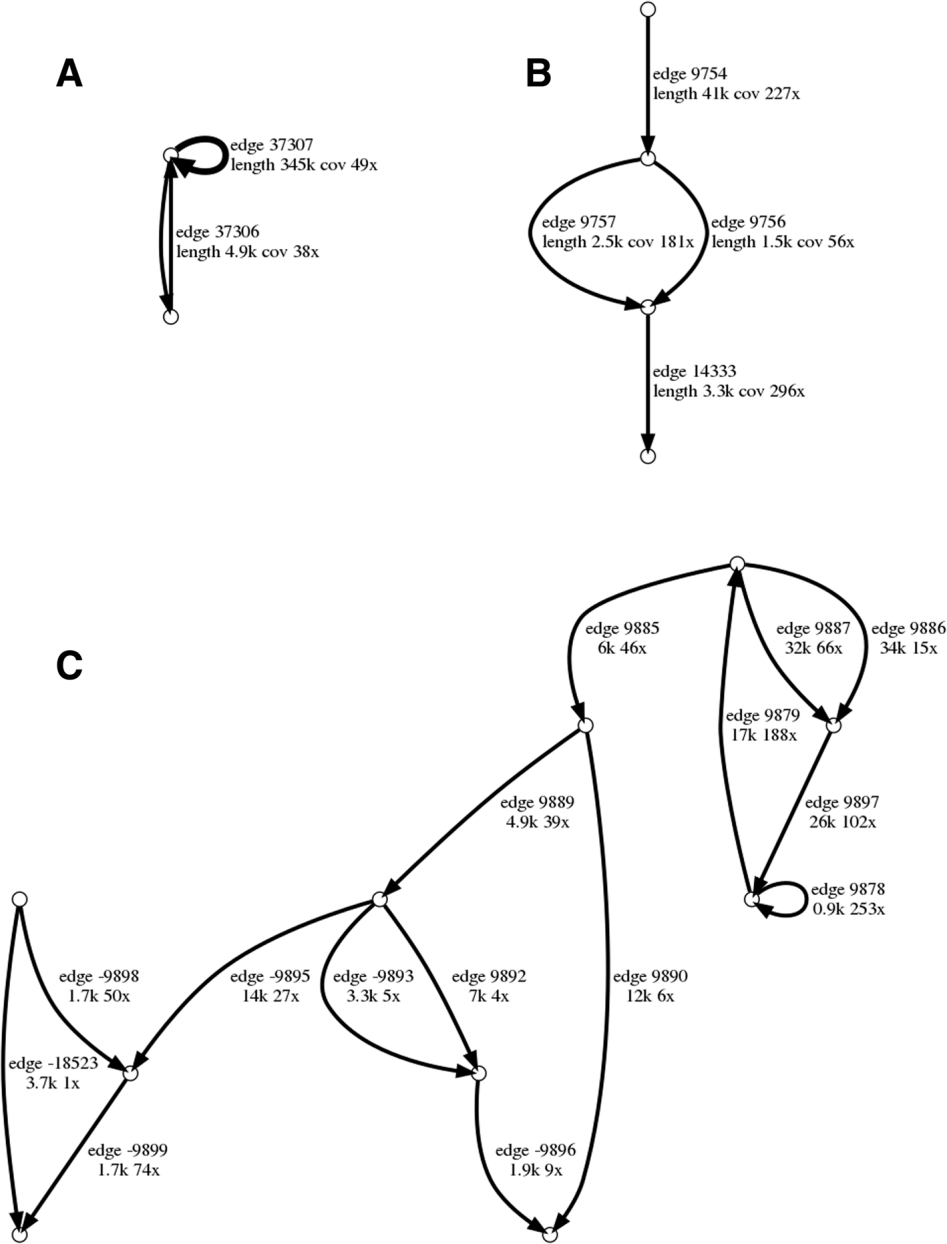
Examples of the multi-edge connected components in assembly graphs in the SHEEP_GUT and ALOHA_VIROME datasets visualized by the AGB tool [47]. (A) A two-edge component in the SHEEP_GUT dataset likely represents a virus with the genome RUR*, where R and R* form a 4.9 kb inverted terminal repeat and U is a 345 kb long unique region. (B) A four-edge component in the ALOHA_VIROME dataset likely represents two viral strains that differ from each other by a diverged region of length 2.5 kb in one virus and of length 1.5 kb in another virus. (C) A more complex viral component with 15 edges (total length 150 kb) in the ALOHA_VIROME dataset

Table 4 presents the benchmarking results on the SHEEP_GUT dataset. viralFlye identified 158 linear viral contigs, 153 circular viral contigs, and 23 multi-edge viral components in the SHEEP_GUT dataset. Additional manual analysis revealed that four of these components represent viruses with inverted terminal repeats and eight likely represents multi-strain viral quasispecies. It remains unclear how to extract viral sequences from the remaining 11 multi-edge components (see Fig. 3 and additional file 1); moreover, some of them may represent assembly artifacts.

**Table 4:** Metagenome-assembled viruses in the SHEEP_GUT dataset identified in various assemblies

Sample	metaFlye +			viralFlye			Raven +		
	viralTools						viralTools		
	Linear	Circular	Components	Linear	Circular	Components	Linear	Circular	Components
SHEEP_GUT	79	185	23	158	153	28	24	22	15
Contigs with “high” or “medium” completeness by checkV	75	181	18	139	150	23	24	22	14

Although the metaFlye pipeline assembled more circular viral contigs, viralFlye assembled more linear contigs—the result of some circular genomes assembled by meatFlye re-classified as linear by viralFlye. We randomly selected 10 such viral contigs and examined their read alignments profiles. In 7 out of 10 cases, the metaFlye contigs were incorrectly circularized due to the chimeric read connections. These incorrectly circularized linear contigs were detected by metaFlye_v and re-classified as linear viruses. In 1 out of 10 cases, the circular contig constructed by metaFlye was likely correct. In the remaining two cases, the manual analysis was inconclusive due to the complex alignment patters.

checkV classified 139 out of 158 linear viral contigs and 150 out of 153 circular viral contigs as complete. The average completeness (estimated with checkV) was 90.7% for linear and 92.8% for circular viral contigs. CircularDisconnector found 16 incorrectly circularized linear contigs in this dataset.

We note that there are 153 high-coverage circular contigs shorter than 1Mb in the metaFlye_v assembly. Since the circular contigs represent isolated loops in the metaFlye_v assembly graph, they likely originate from viral genomes and plasmids. Among 55 non-viral circular contigs, 20 were identified as plasmids using the plasmid identification approach from [31] and 9 as bacterial and eukaryotic contigs, including the complete mitochondrions of the sheep host (*Ovis aries*) and a sheep parasite *Blastocystis spp.*. The remaining 26 uncharacterized circular contigs may represent novel and unusual plasmids and circular viruses that have evaded identification by the existing plasmid and virus identification tools (although some of them may represent assembly artifacts).

On the other hand, metaFlye_v generated 158 non-characterized high-coverage linear contigs in the SHEEP_GUT dataset. These contigs likely originate either from novel and unusual linear viruses or from bacterial genomes with highly non-uniform coverage that resulted in sharp coverage drops. Figuring out which of the uncharacterized circular and linear contigs represent new viruses remains an open problem.

### CRISPR-based host prediction

Identification of known CRISPR spacers in MAVs is a powerful approach for predicting potential known hosts for novel viruses from metagenomic assemblies [1, 32]. However, this approach is limited by the CRISPR arrays available in the existing (rather incomplete) CRISPR databases. Here, we demonstrate that the novel CRISPR arrays identified in a newly assembled metagenomic sample provide additional and more accurate information for predicting potential novel hosts for novel viruses. We focused on this database-free “novel host-novel virus” mode and compared it with the traditional database-based “known host-novel virus” approach. For the SHEEP GUT dataset, where both long and short reads (with the high coverage) were available, we studied intra-sample virus-host associations for both types of data, and compared them with the results obtained using the CRISPRCasdb database (see the “Methods” section for details).

Illumina reads from the SHEEP_GUT dataset were assembled using MEGAHIT v1.2.9 [9]. PacBio HiFi reads from the SHEEP_GUT dataset were assembled using viralFlye [24]. To predict potential viruses, we extracted all circular and linear MAVs in both short-read and long-read assemblies. Interestingly, the sets of MAVs identified in short-read and long-read assemblies were quite different with only 85 MAVs shared between two assemblies (Table 5).

**Table 5:** Information about the virus-host assignments derived by analyzing the CRISPR spacers

	Total	MAVs with predicted hosts (derived by searching for potentially novel CRISPR arrays in the assembly)	MAVs with predicted hosts (derived by matching against known CRISPR arrays in CRISPRCasdb)	MAVs identified by both approaches
MAVs identified from the long-read dataset (PacBio HiFi)	339	56 (16.5%)	74 (21.8%)	9 (2.6%)
MAVs identified from the short-read dataset (Illumina)	303	15 (5%)	54 (17%)	2 (0.6%)
MAVs shared between the long-read and short-read datasets	85	Long reads: 10 (11%)	17 (20%)	3 (3.5%)
		Short reads: 7 (8.2%)		1 (1.1%)

The host contigs in the long-read assembly were significantly longer (average/median length 519/135 kb in the long-read assembly as compared to 13/3.5 kb in the short-read assembly). Interestingly, although matching against the CRISPRCasdb library identified more virus-host pairs, these predictions show a very small overlap with the identified intra-sample virus-host pairs. Thus, complementing the traditional matching against the CRISPR database with the intra-sample host predictions significantly increases the number of the predicted virus-host associations.

In most cases, the host taxonomy in both datasets and the CRISPRCasdb library was in agreement, at least at the order level (Additional file 3: Table S1). In the case of long-read contigs, the taxonomic position of the potential hosts can be determined with a higher precision—hosts for 14 out of 15 viruses were predicted up to the family level (5 out of 8 viruses in the MEGAHIT assembly), in large part because of the fact that the longer contigs allow one to better identify the matching bacteria.

## Discussion

Although the set of known viral genomes has been steadily expanding, only a tiny fraction of the Earth’s virome has been sequenced so far. The recent long-read metagenomic studies have generated assemblies that greatly improve over the short-read assemblies with respect to assembling complete and nearly complete bacterial genomes and opened the era of “complete metagenomics” [24, 25]. However, the algorithms for generating and analyzing MAVs in long-read assemblies remain underexplored. In the absence of benchmark datasets with known references (such as the HMP mock and SYNTH datasets for bacterial metagenomes [33]), comparing different assembly methods is a difficult task. Nevertheless, we have shown that viralFlye improves reconstruction and analysis of MAVs as compared to the previously described approaches [14, 15, 27] to MAV identification in long-read assemblies.

We showed that the previously developed custom pipelines for MAV generation may lead to both missing and incorrectly assembled MAVs. In the case of the HUMAN_GUT dataset, viralFlye identified 5 times more MAVs than the pipeline in [15]. In the case of the ALOHA_VIROME dataset, viralFlye increased the number of identified MAVs by 63% as compared to the pipeline in [27] (across all three samples). Interestingly, viralFlye revealed many multi-edge viral components in long-read assemblies (across all datasets) that either represent viruses that have long repeats or a population of multiple viral strains (species) that have structural variations or highly diverged regions. Since our knowledge of the diversity and frequency of structural variations in viruses is limited [34], decomposing these viral components into individual strains would contribute to evolutionary studies of viral populations. However, dissecting these viral components into individual strains represents an open computational problem. Since viruses forming multi-edge connected components remained below the radar of previous studies, solving this problem will likely reveal many previously unknown viruses and will provide insights into how multi-strain viral communities are organized and how they evolve.

Although identification of *known* CRISPR spacers in MAVs is a widely used approach for predicting potential *known* hosts for *novel* viruses, this *sample-independent* approach does not analyze bacteria present in the sample and is not applicable to still unknown CRISPR spacers. We demonstrate that the identification of novel CRISPR arrays in bacterial genomes from a newly assembled metagenomic sample (*sample-dependent* approach) provides information for predicting potential *novel* hosts for *novel* viruses and results in a large number of identified virus-host associations. Interestingly, there is a very small overlap between the viral-host pairs identified by the sample-dependent and the sample-independent approaches, suggesting that these approaches are complementary.

Our study revealed 1036 uncharacterized circular (15967 linear) contigs which can originate from novel and unusual viruses and plasmids. There are multiple reasons that may explain a large number of uncharacterized potentially viral contigs in our assemblies. First, since the existing viral identification tools were trained on known viruses, they are unlikely to classify a contig as viral if it represents a highly diverged virus. Second, since the Stop codon reassignment is common [3, 35], these tools may mispredict genes and again fail to characterize a contig as viral. Third, some of the uncharacterized contigs may represent assembly artifacts or represent contigs with basecalling errors that affected gene prediction and downstream viral identification. Thus, identifying uncharacterized contigs that originate from viral genomes remains an open problem that we plan to address in a follow-up study.

## Conclusions

Although metagenomic sequencing has greatly expanded our knowledge of the Earth’s virome, extracting complete sequences of viral genomes from metagenomic assemblies remains challenging. Previous studies, aimed at the discovery of novel viruses, often focused on viral contigs in metagenomic assemblies and thus missed an opportunity to sequence complete viral genomes by switching from the contig-based to the assembly graph-based analysis. Emergence of long reads opened a possibility to sequence many complete viral genomes that evaded all attempts to sequence them using short reads.

We demonstrated that viralFlye improves identification of complete viruses from long-read metagenomic datasets and has a potential to transform metagenomics-based assembly of novel viruses from a challenging task into a routine procedure. viralFlye recovers up to 2.25 times more complete or nearly complete MAVs, as compared to the previously published pipelines, while reducing the number of misassemblies. We also show that long reads improve the accuracy of virus-host association predictions and reveal many viral quasispecies, populations of multiple viral strains (species) that have structural variations or highly diverged regions.

## Methods

### Modifying metaFlye for viral assembly

metaFlye generates *disjointigs* and “glues” them into the metagenome assembly graph [24]. This procedure typically results in generating many short disjointigs (with length comparable to the length of a single read), most of which represent assembly artifacts rather than real biological sequences. However, some of them may represent short plasmids and viruses. Since it is unclear how to separate artifacts from viral and plasmid contigs, metaFlye (that was originally designed for bacterial assemblies) discards many short disjointigs, thus filtering out short viral disjointigs. Another limitation of metaFlye is that it may connect two or more linear viruses in a single chimeric MAV, an artifact caused by chimeric reads formed by concatenating segments from multiple viruses. It turned out that chimeric reads are often formed by concatenating the ends of two different viral genomes.

To address these complications, we modified the disjointing assembly algorithm in metaFlye by (i) preserving rather than discarding short disjointigs derived from reads covering the entire viral genome and (ii) aggressively filtering possibly chimeric reads that resulted from multiple linear viruses. To enable (i), in the updated metaFlye_v tool, we allow a disjointig to be represented by a single read, provided that such read is supported by other reads. To enable (ii), metaFlye_v detects ends of linear viruses characterized by a sharp drop in coverage by reads; such regions are then subjected to stricter parameters for the chimera detection algorithm [24]. Applying this approach led to a significant reduction in misassemblies, e.g., by an order of magnitude on the WARWICK_VIROME dataset.

### Polishing the metaFlye assembly with short reads

In the case when both long error-prone reads and short accurate (Illumina) reads are available, viralFlye polishes the metaFlye long-read assembly using short reads. The polishing step reduces the number of base-calling errors and indels in metaFlye assemblies of long error-prone reads (particularly in the case of low-coverage genomes) and thus improves gene prediction, an important step for the success of the downstream virus identification tools [10, 29]. viralFlye uses the *freebayes* tool [36] (with the bwa mem aligner [37]) followed by the *bcftools consensus* tool [38] for polishing.

### Identifying isolated viral contigs in the assembly graph

There exist multiple viral detection tools that classify each contig into viral and non-viral, such as viralVerify [10] and checkV [29]. viralFlye analyzes the extracted contigs using the viralVerify v1.1 tool [10] with default parameters to select contigs representing putative viral sequences. Since some contigs may represent partial rather than complete viral genomes, we have additionally checked the completeness of linear and circular contigs using the viralComplete tool [10]. Since viralComplete evaluates the completeness of a viral genome based on its similarities to known viral genomes (using the *completeness* threshold), it often underestimates the completeness in the case of novel viral genomes with limited similarities to known viral genomes. Since metagenomic samples likely contain many viruses without close database references, we have reduced the completeness threshold to 50% and provided an option to change it.

### Identifying multi-edge viral components in the assembly graph

Even though most viral genomes are much shorter than bacterial genomes, environmental viral samples often have high inter- and intra-species heterogeneity [39]. This heterogeneity leads to fragmentation of both short-read and long-read metagenomic assemblies [24, 33]. Ideally, complete viral genomes are represented as isolated edges (linear viruses) or isolated loops (circular viruses) in the metagenomic assembly graphs [15, 20]. However, as we have shown in the “Results” section, many viruses form more complex multi-edge connected components in the long-read assembly graphs. The ComponentTraverser module of the viralFlye pipeline analyzes such components and identifies the components that are likely formed by viral genomes.

A connected component of an assembly graph is called *viral* if it represents a viral genome (possibly with the addition of a small number of edges from non-viral genomes). Although there exist multiple viral detection tools that classify isolated contigs into viral and non-viral ([10, 29]), classification of multi-edge connected components of an assembly graph into viral and non-viral is an open problem.

We define the *length* of a connected component *Component* in an assembly graph (denoted as *length*(*Component*)) as the total length of its edges. A connected component is called *small* if *minLength*<*length*(*Component*)<*maxlength*, where *minLength*, and *maxLength* are thresholds with default values *minLength*=5 kb and *maxLength*=1 Mb (length of most viruses falls into this range). The coverage of a connected component *Component* is defined as the average coverage of its edges normalized by the edge length, i.e, as

$\sum _{each\ edge\ e\ in\ Component} {coverage}({e}) \cdot {length}({e})/{length}({Component)}$

The ComponentTraverser module classifies each small high-coverage connected component of the assembly graph as either viral or non-viral. Given a connected component, it generates random traversals of this component and then launches a viral detection tool on each random traversal. To generate a random traversal, ComponentTraverser launches a random walk that starts from a path formed by a single edge (the longest edge in the component) and gradually extends this path in both directions by randomly adding still untraversed edges until no such edges are found. ComponentTraverser generates *numberTraversals* such paths (default value 10).

viralFlye classifies a multi-edge component as viral if one of the generated traversals is classified as viral by a viral detection tool. It further reports all multi-edge viral components in individual files for further manual examination. Figure 3 shows some examples of viral multi-edge components found by ComponentTraverser.

### Classifying MAVs into linear and circular

Many linear viruses have *direct terminal repeats* (*DTRs*) on both ends that can span 10 kb and more in length [40]. Since linear viruses with long DTRs are typically assembled into a loop in the assembly graph, they are often misclassified as circular viruses. Although this misclassification is particularly rampant in short-read assemblies [41], it extends to long-read assemblies in the case when the DTR length is similar or longer than the typical read length. For example, as shown in [15], five viruses from the crAssphage family reported as circular MAVs in the previous short-read study [42] represent linear MAVs. The CircularDisconnector module of viralFlye checks each circular viral contig in the assembly graph and attempts to figure out whether it is in fact a linear contig “glued” into a circular contig by DTRs.

CircularDisconnector is based on the observation that the two DTR regions in a linear genome (at the beginning and at the end of the genome) are “glued” into a single region with sharply increased coverage (as compared to the average coverage of this genome) in the assembly graph. The coverage of this region is expected to be nearly twice larger (as compared to the average coverage of the viral genome) because it is covered by reads from both the beginning and the end of the genome.

**Fig. 4:**
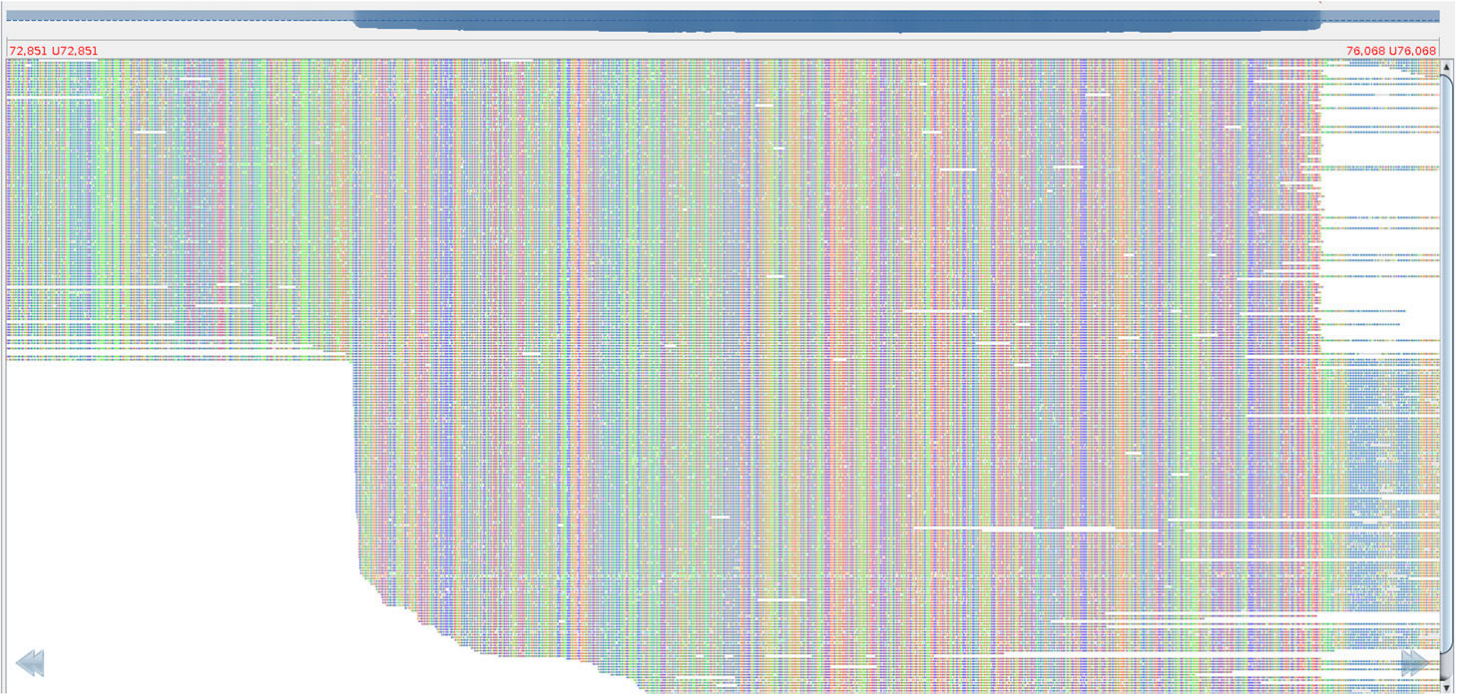
Alignment of long reads from the sample apr34 to crAssphage apr34_000142F genome (MK415399.1) analyzed in [15]. The alignments of reads covering the region (from positions 72850 to 76060) that contain the DTR location of length 2 kbp (positions from 73630 to 75800). There is a roughly double increase in coverage in the DTR, and only two reads that traverse through this DTR. The figure was generated using the Tablet visualization software [48]

A position in a contig is classified as a coverage *jump* (coverage *drop*) if its coverage is larger (smaller) than the coverage of the neighboring 100 bp long region by a factor of *CoverageJump* (default value *CoverageJump*=1.5). To decide whether a circular contig represents a linear genome, CircularDisconnector checks whether there exist coverage jumps/drops in this contig. Figure 4 shows an example of an increased coverage in the DTR region that reveals one coverage jump and one coverage drop.

Another indication that a region between two positions in a circular contig actually represents a DTR in a linear virus is the sharp drop in the number of reads spanning these two positions (as compared to the average number of reads spanning segments of the same size). Although we do not expect to see any reads spanning both these positions of a linear genome (and extending beyond them), (i) chimeric reads, (ii) reads arising from a linear genome in the lysogenic stage, and (iii) reads arising from a linear genome concatenation during the *rolling circle replication* may span these positions. However, since the number of such reads is typically small, we classify a region between two positions as a *low-coverage region* if the number of reads spanning both these positions is at least *coverageReduction* times lower than the average number of reads spanning regions of the same size in the assembled genome (default value *coverageReduction*=10).

CircularDisconnector examines each circular viral contig in the metaFlye assembly, aligns all reads to this contig, and detects all coverage jumps/drops using the *sliding window technique* [43]. It further identifies contigs with a single coverage jump and a single coverage drop and analyzes them as potential indicators of the DTR gluing in assembly graphs of linear genomes. It classifies a contig as linear if the identified coverage jump and coverage drop (located at positions *jump* and *drop* separated by distance *span*=|*drop*−*jump*|) satisfy the following two conditions: (i) the region between positions *jump* and *drop* is a low-coverage region and (ii) *span*>*minSpan*. The default value of *minSpan* equals the parameter *min-overlap* (default value 1000 bp) in metaFlye that defines the minimum overlap between disjointigs in the metaFlye gluing procedure (overlaps smaller than *minSpan* are not glued by metaFlye).

### CRISPR-based prediction of virus-host associations

The standard approach for predicting potential *known* hosts for *novel* viruses from metagenome assemblies is based on identifying known CRISPR spacers in MAVs [1, 32]. It is thus limited by the CRISPR arrays available in the existing (rather incomplete) CRISPR databases. The “Results” section demonstrates that the novel CRISPR arrays identified in a newly assembled metagenomic sample provide additional and more accurate information for predicting potential *novel* hosts for *novel* viruses.

Given a metagenomic dataset, viralFlye considers all high-coverage circular MAVs, linear MAVs, and multi-edge viral components in its assembly graph (with length varying from 5 kbp to 1 Mb), predicts candidate MAVs using viralVerify, and checks them for completeness using viralComplete (at least 50%). Afterward, it predicts the CRISPR arrays in this assembly using the Minced v. 0.4.2 tool (based on the CRISPR Recognition Tool [44]) and aligns the predicted spacers against all selected MAVs (with parameters max evalue 1E-5, min identity 0.9, blastn-short). The resulting alignments reveal putative virus-host associations. To assess how many of these associations represent known versus novel virus-host associations, the spacers from the CRISPRCasdb database ([45], accessed Nov 5, 2020) are aligned to the predicted MAVs with the same parameters.

## Supplementary Information

**Additional file 1** AGB vizualization of all viral components from SHEEP_GUT dataset.

**Additional file 2** Information about all default settings and versions used for each software tool.

**Additional file 3** **Table S1.** Results of CRISPR-based host prediction results for viralFlye and Megahit assemblies on SHEEP_GUT dataset. **Table S2.** Results of CRISPR-based host prediction results for viralFlye assemblies on WARWICK_VIROME datasets. **Table S3.** Results of CRISPR-based host prediction results for viralFlye assemblies on ALOHA_VIROME datasets. **Table S4.** Results of CRISPR-based host prediction results for viralFlye assemblies on HUMAN_GUT datasets.

**Additional file 4** Review history.

## Data Availability

Source code and scripts for analysis are available on github (https://github.com/Dmitry-Antipov/viralFlye[46]) and zenodo (doi:10.5281/zenodo.5722079) under the BSD license. All the datasets used for the software evaluation are previously published in [6, 15, 24, 27]
