# Supplementary figures and images for: viralFlye: assembling viruses and identifying their hosts from long-read metagenomics data
Source: Genome Biol. 2022 Feb 21;23:57. doi: 10.1186/s13059-021-02566-x (PMC8862349; doi:10.1186/s13059-021-02566-x)

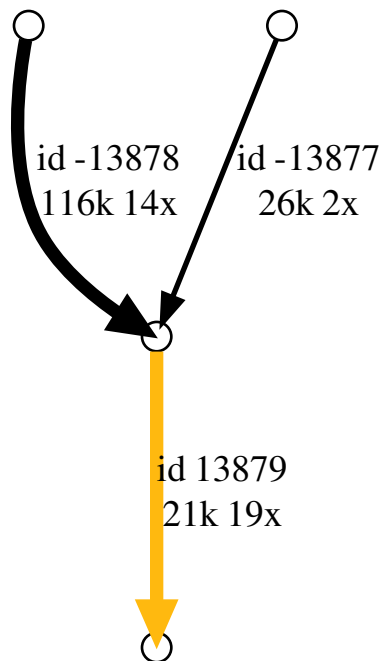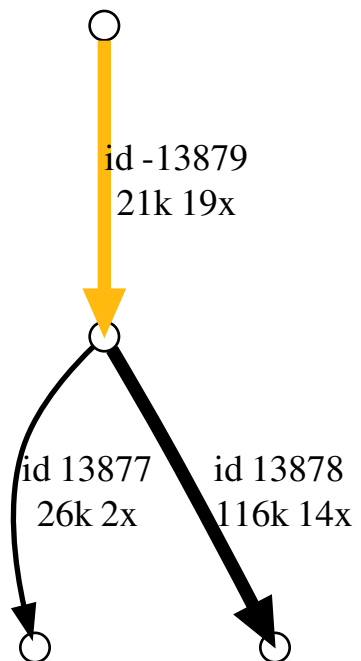

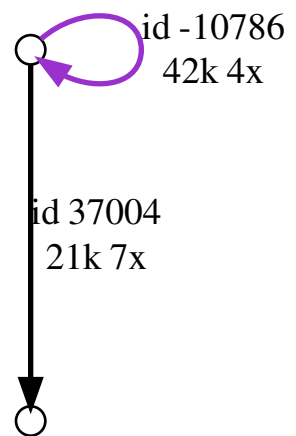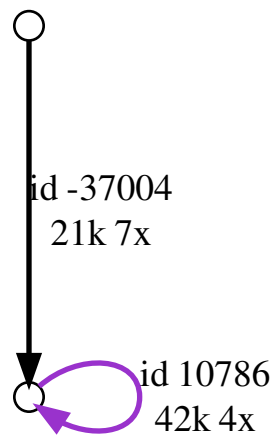

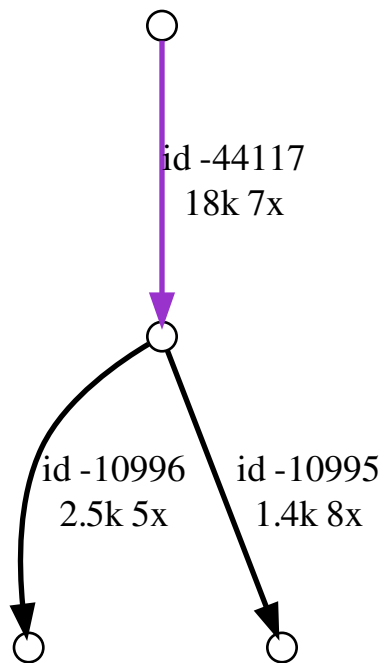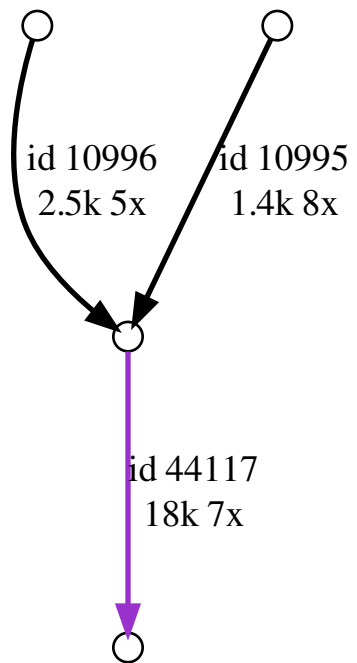

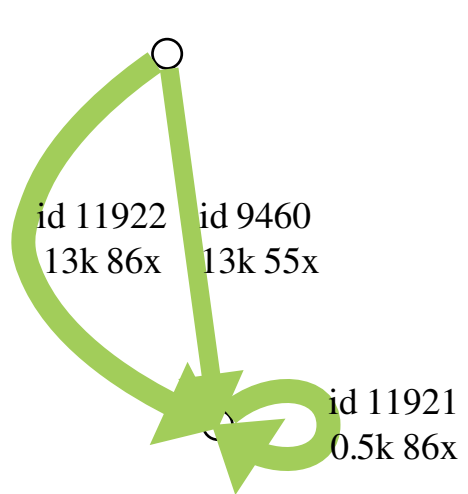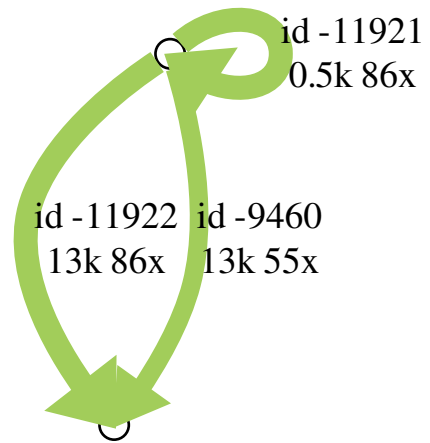

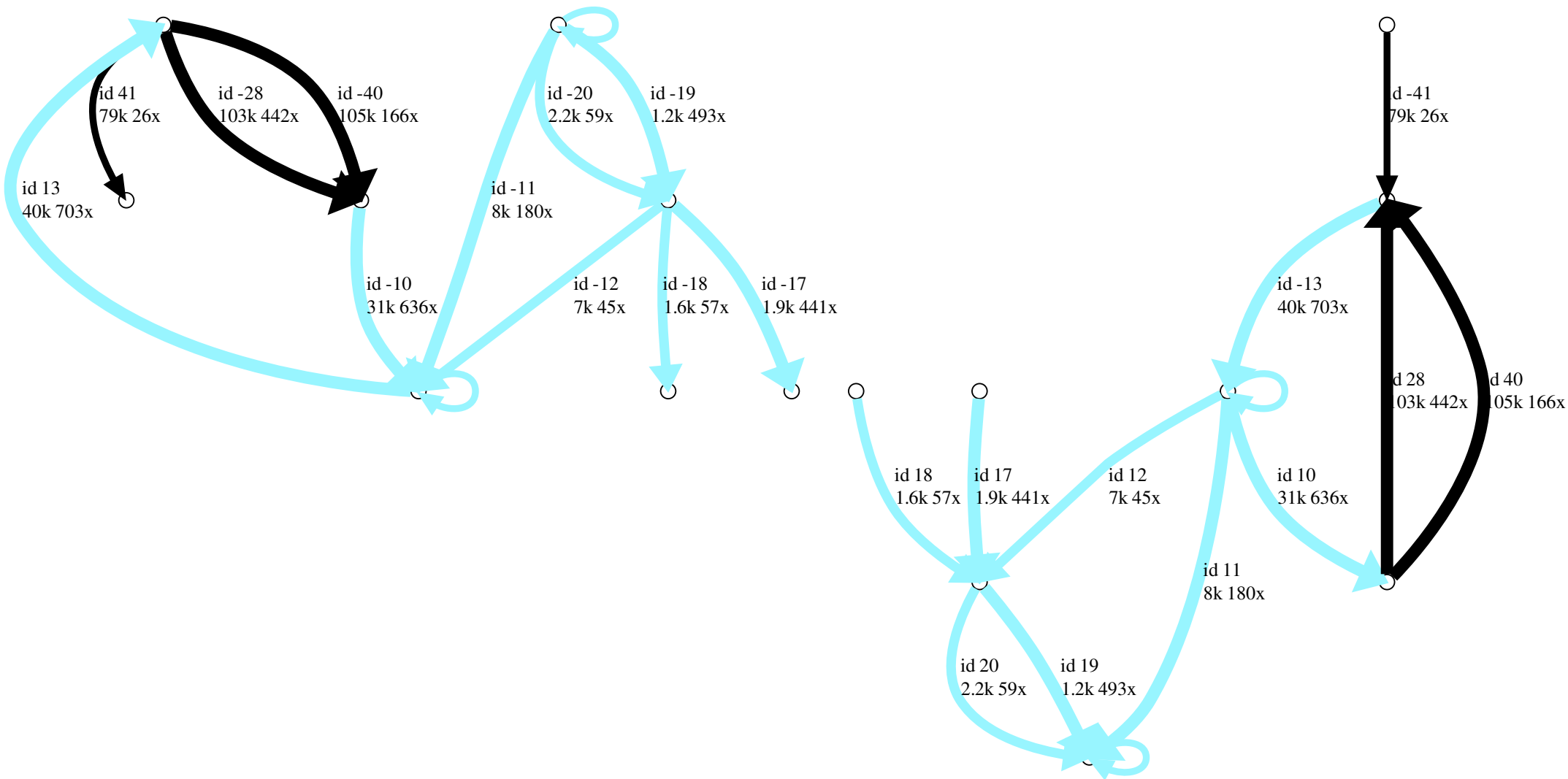

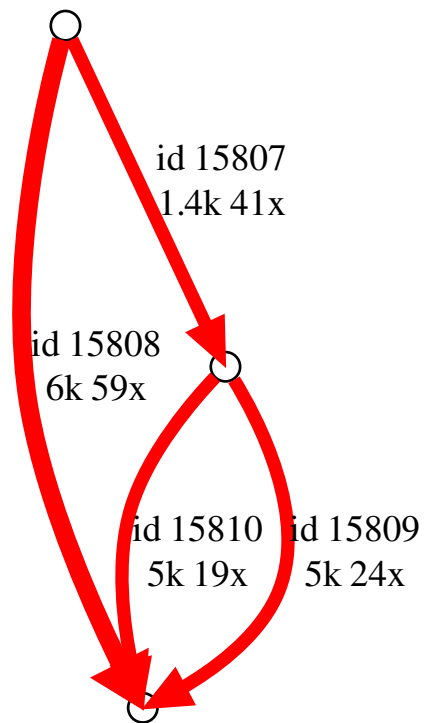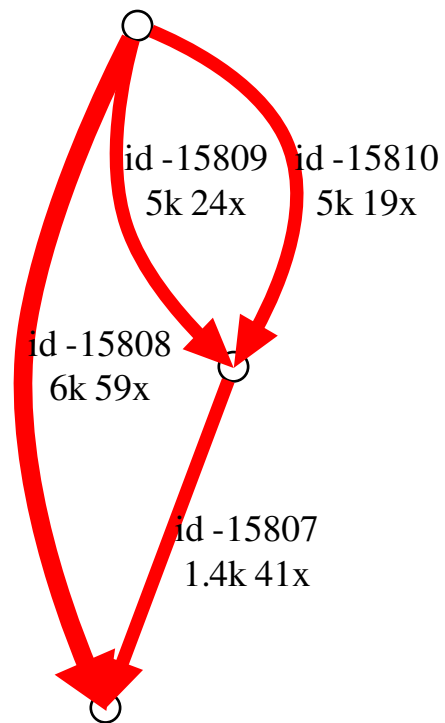

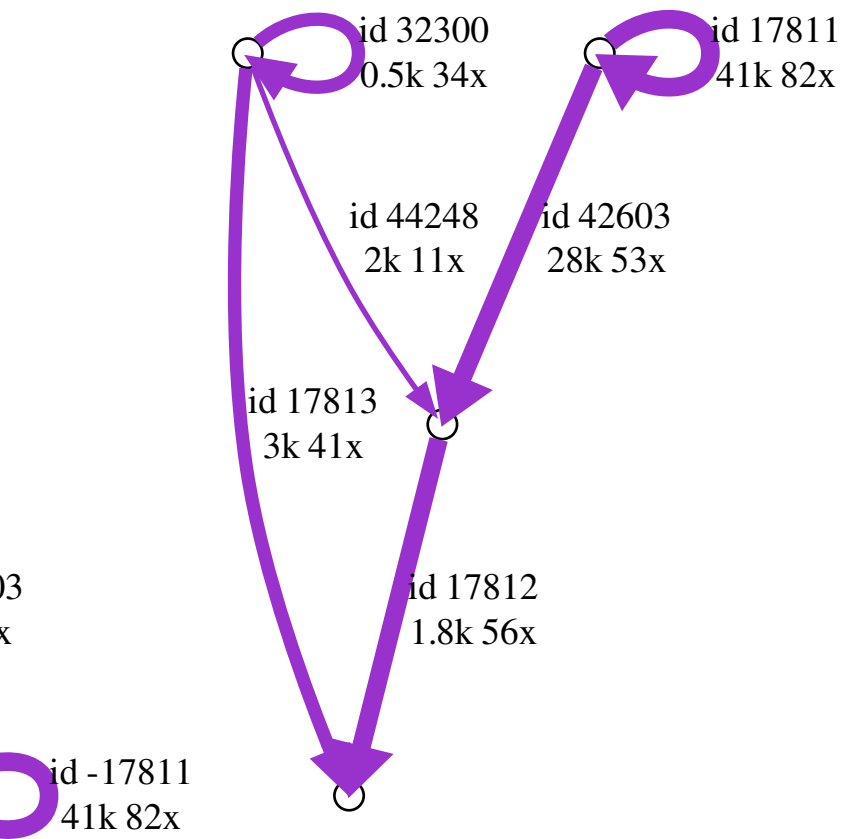

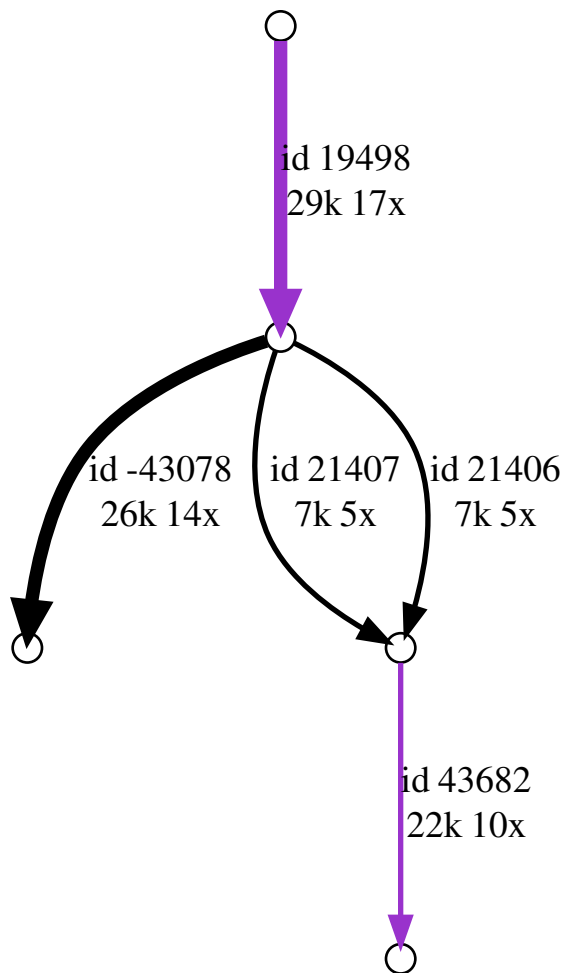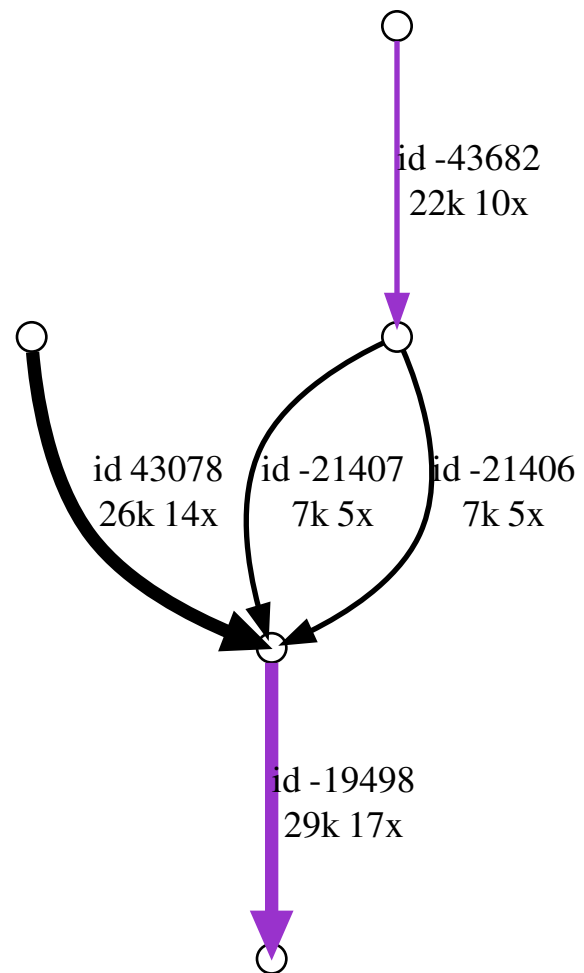

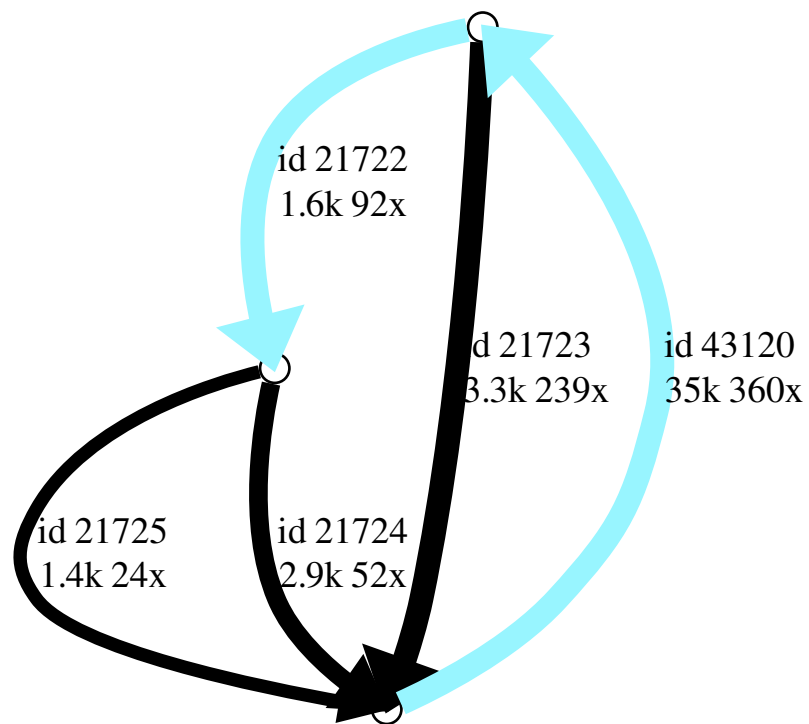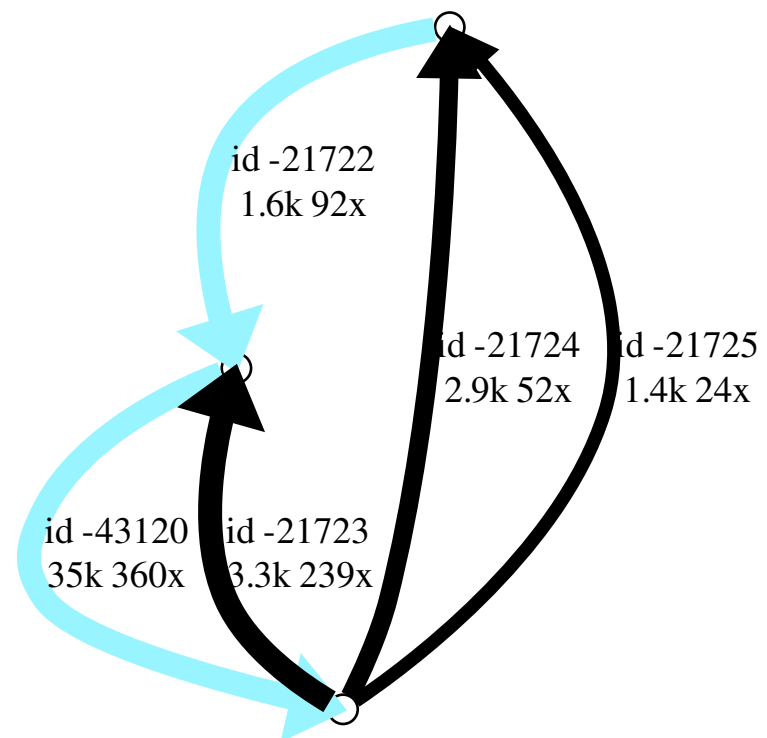

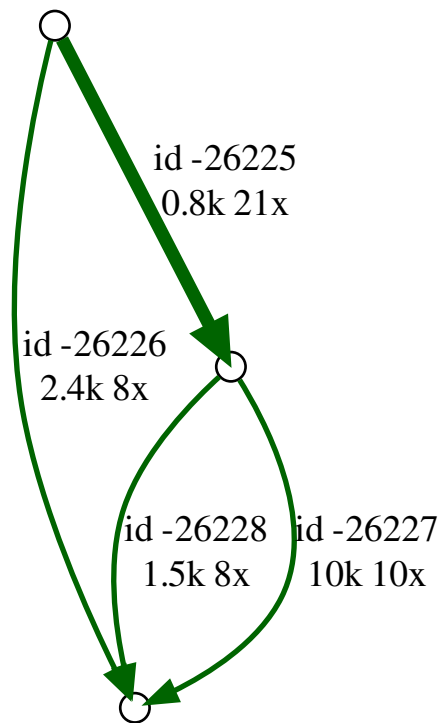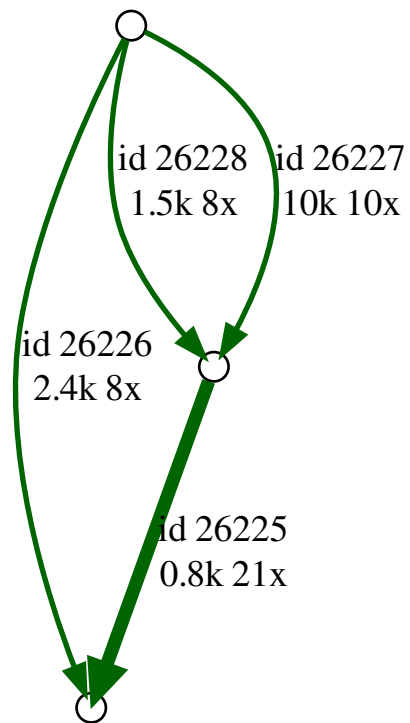

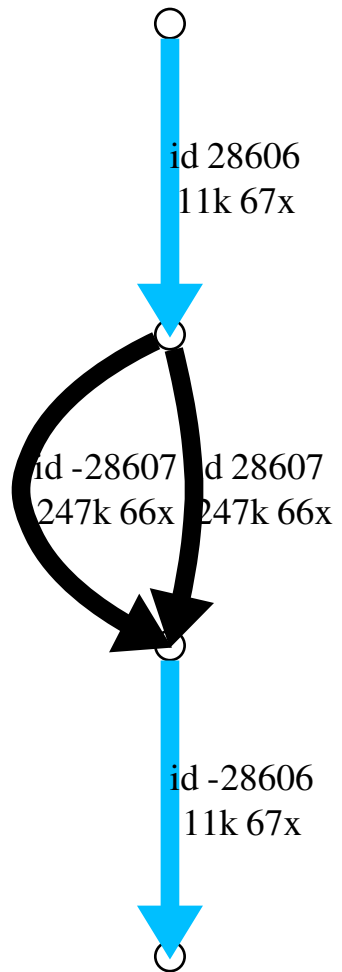

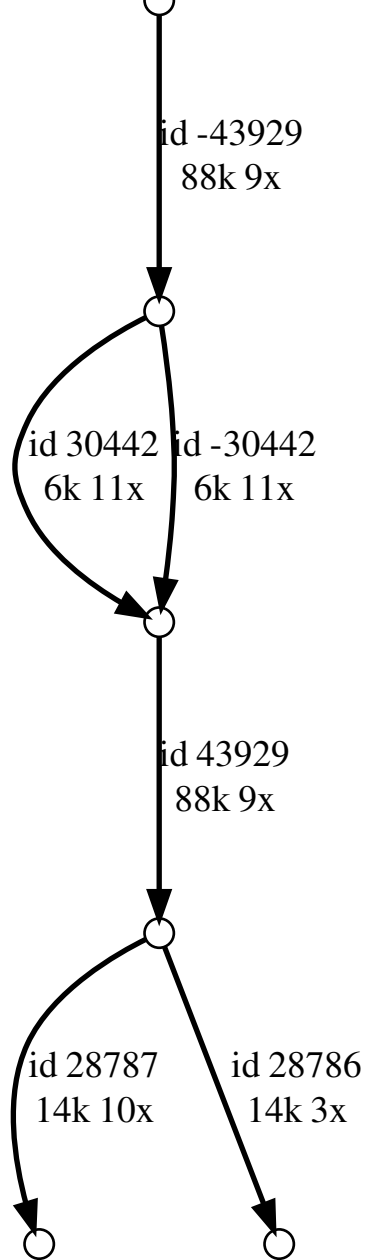

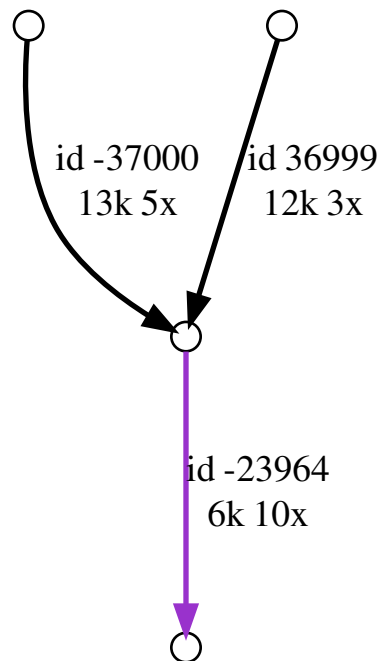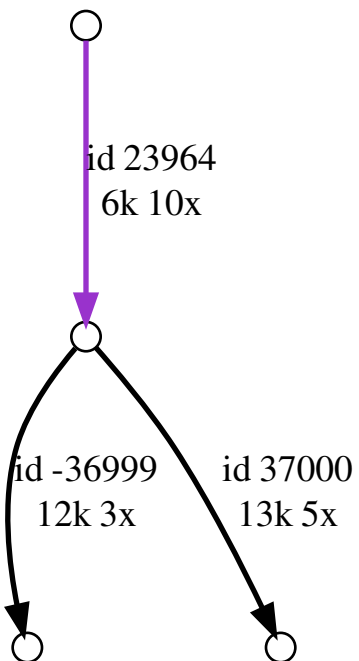

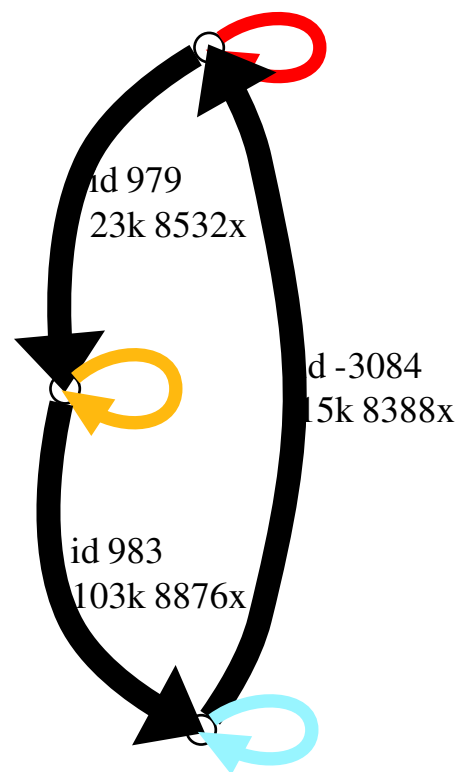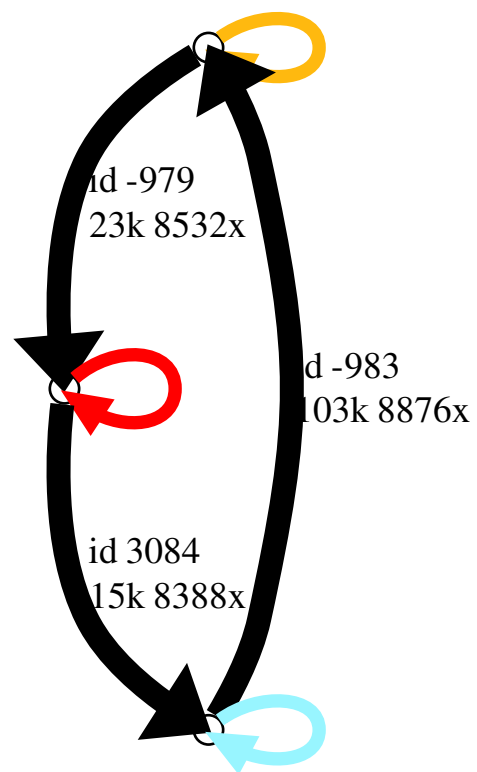

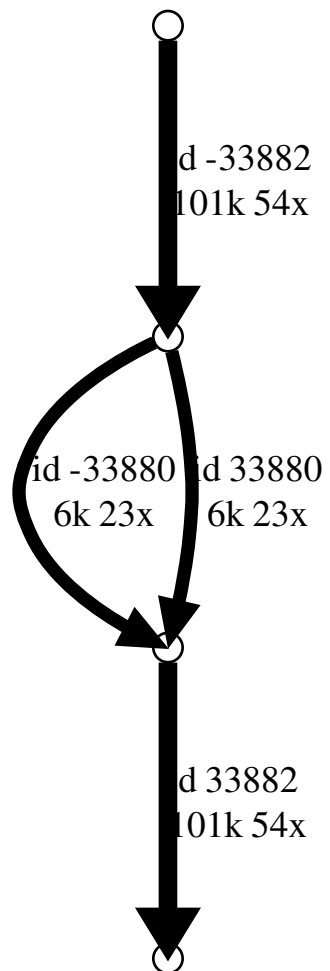

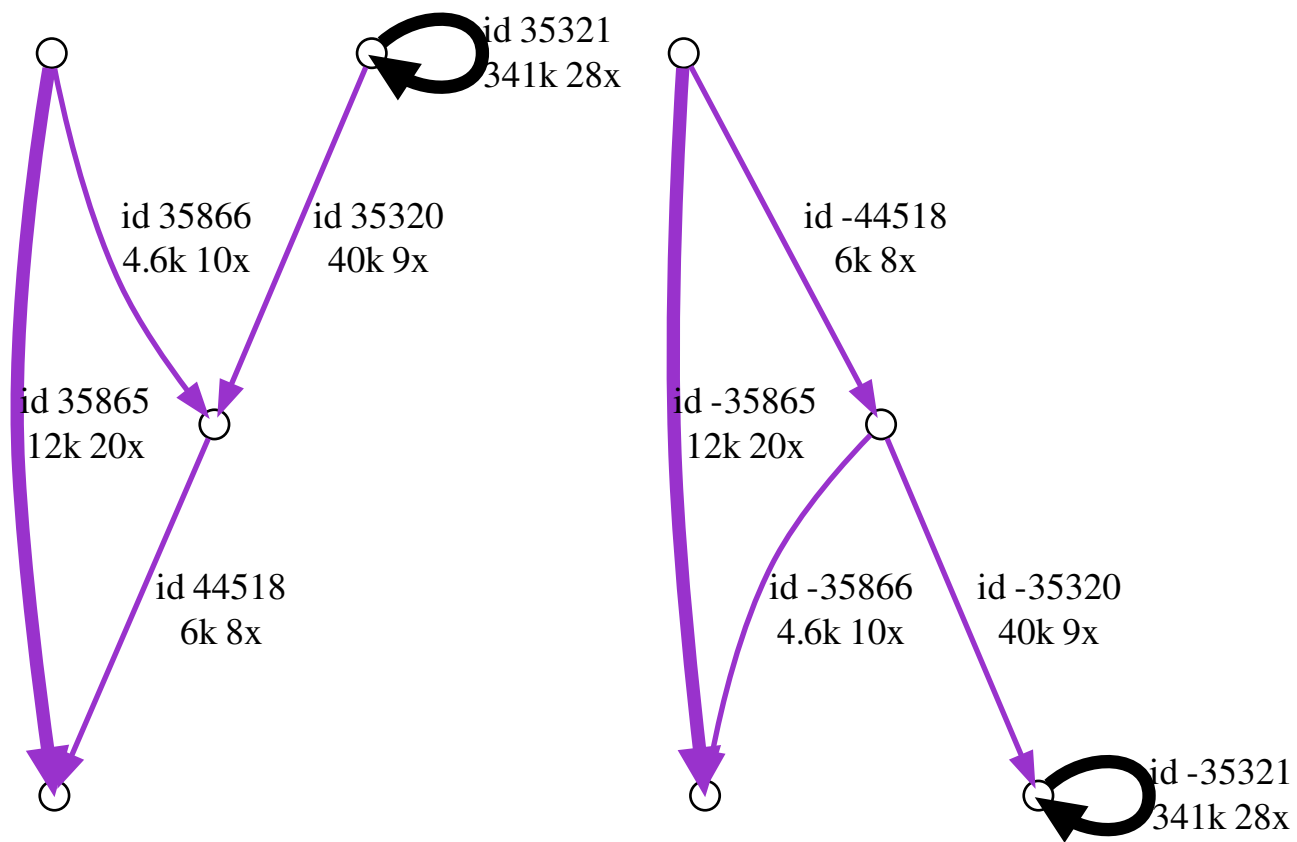

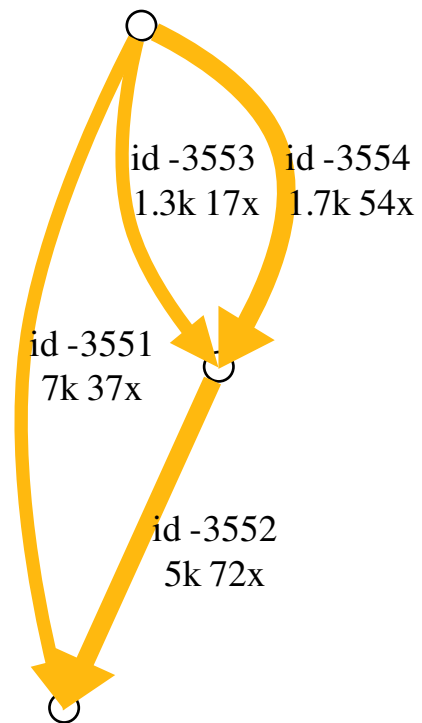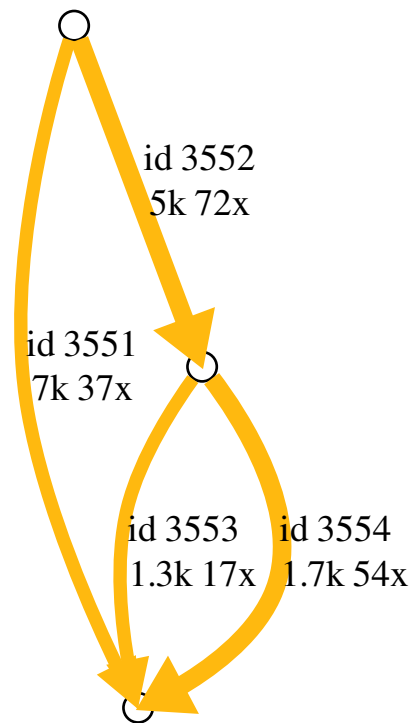

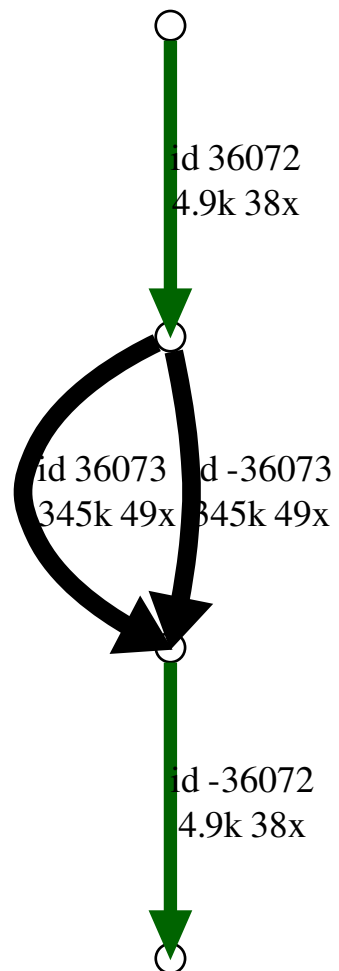

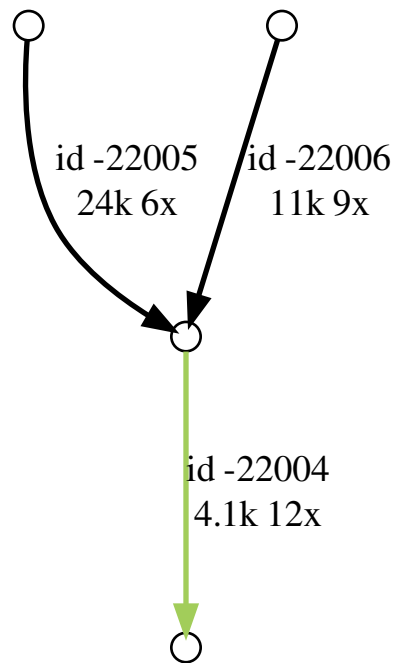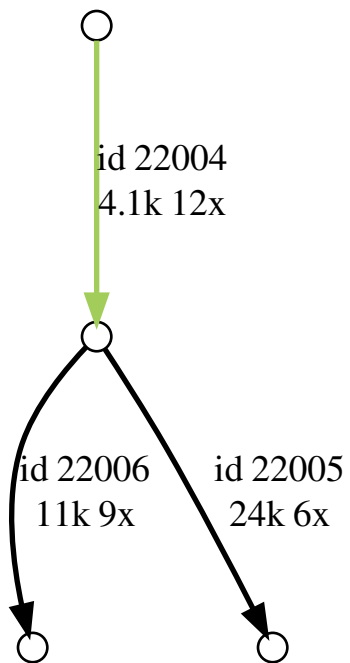

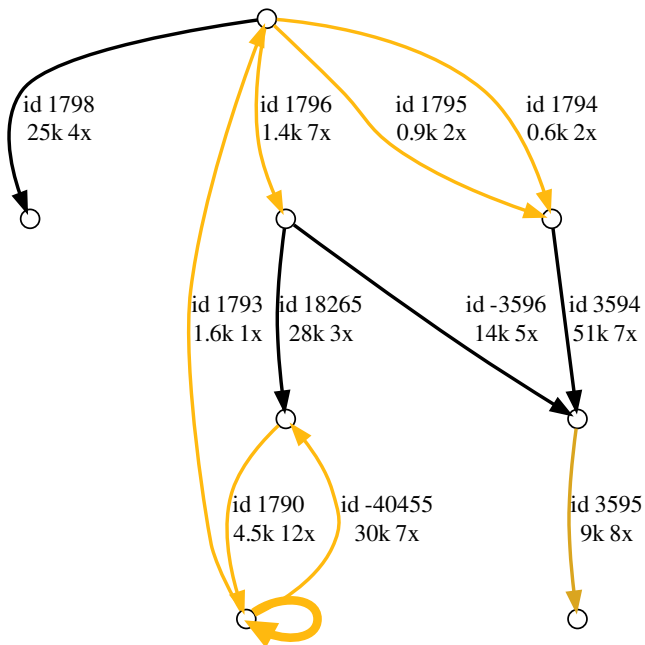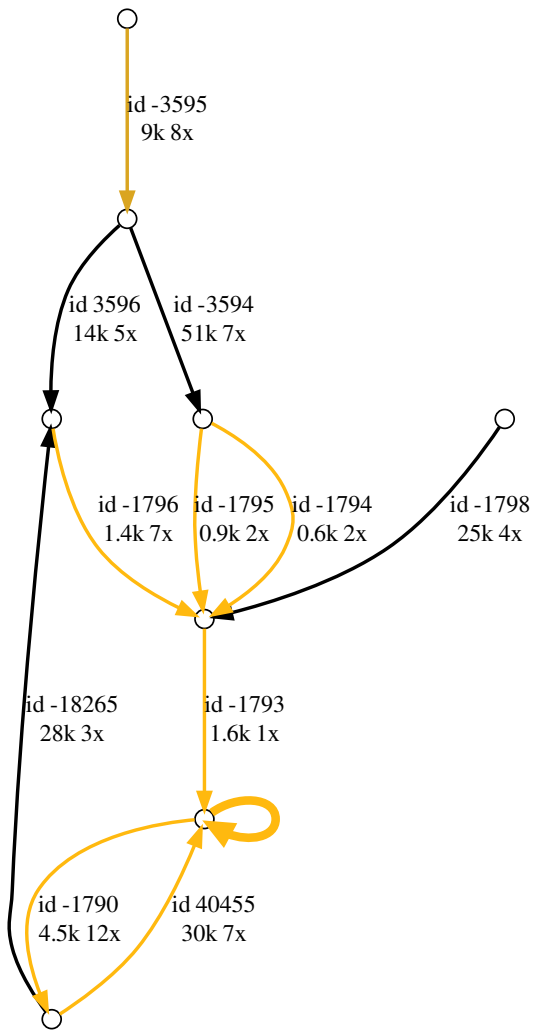

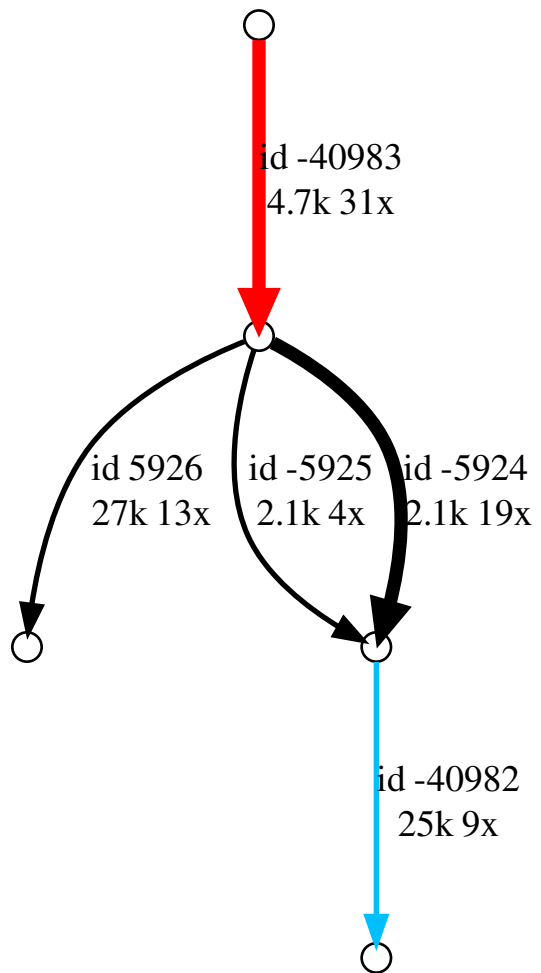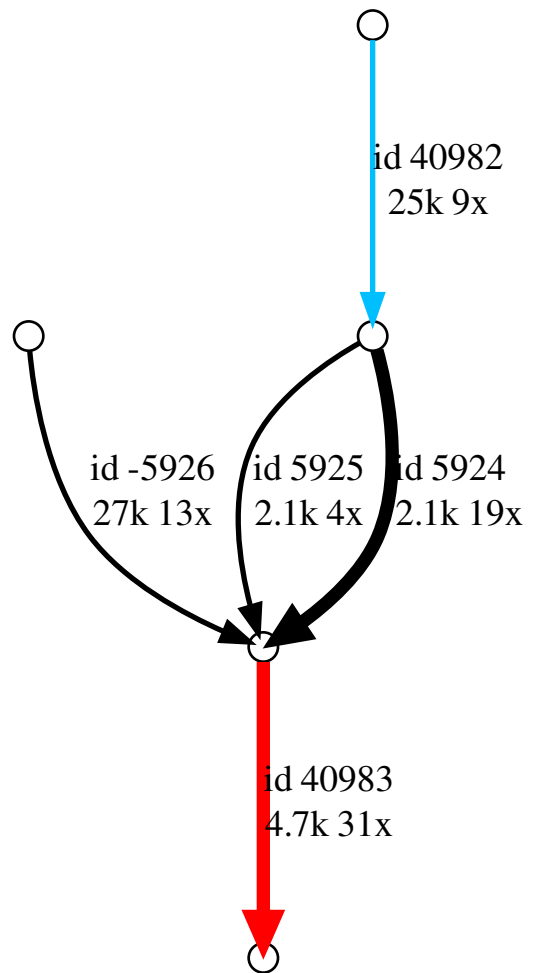

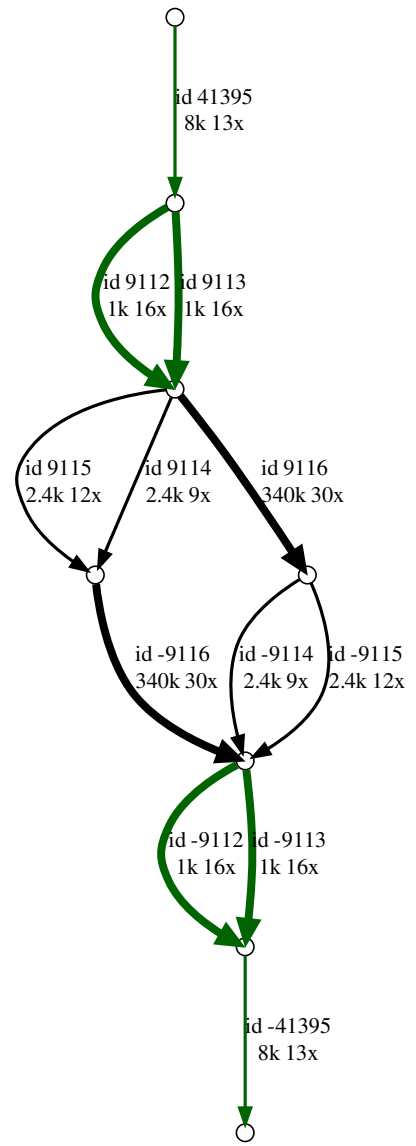

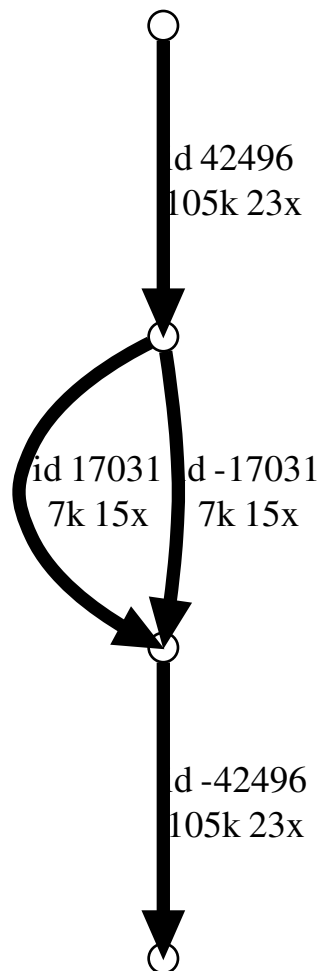

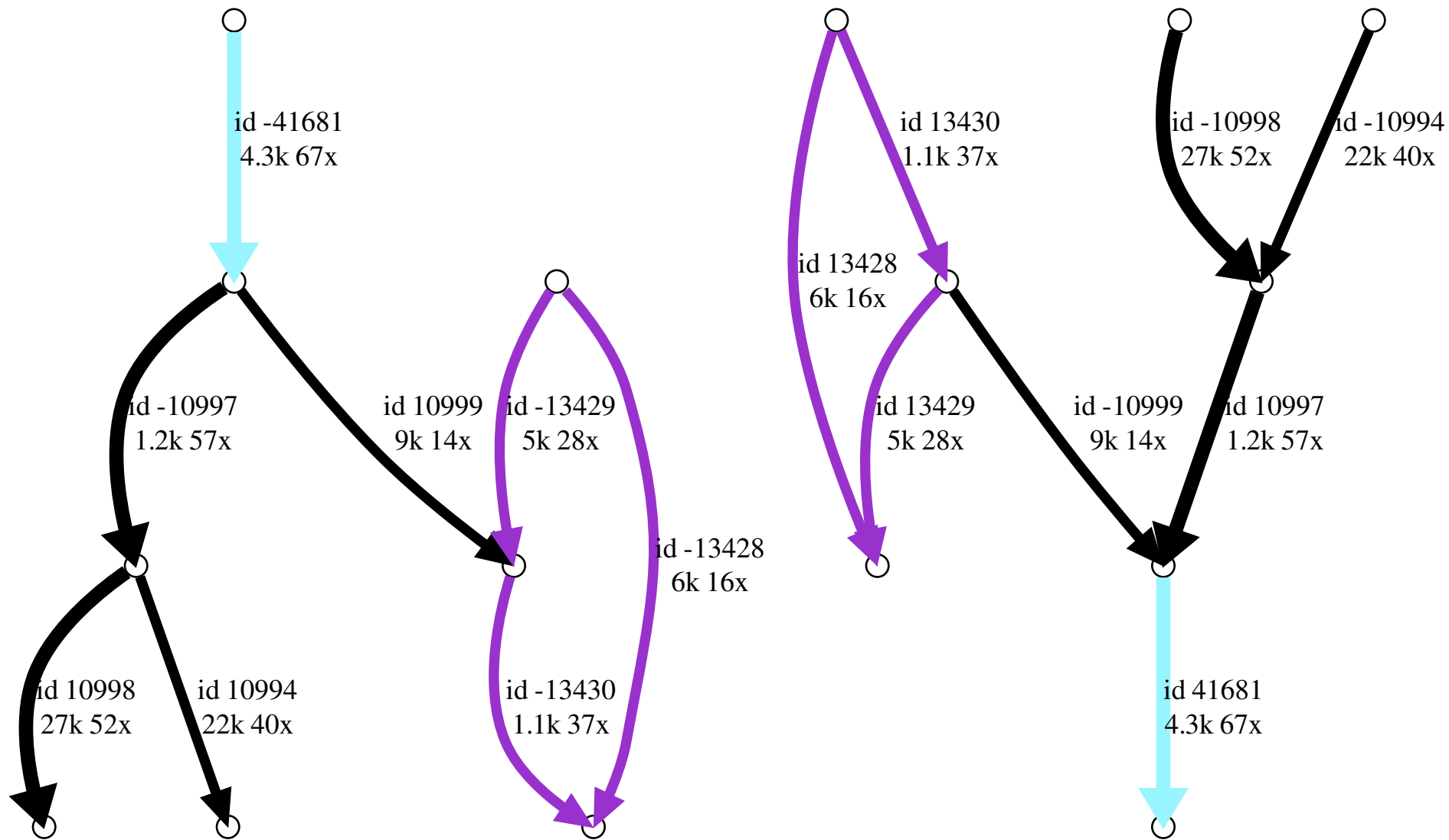

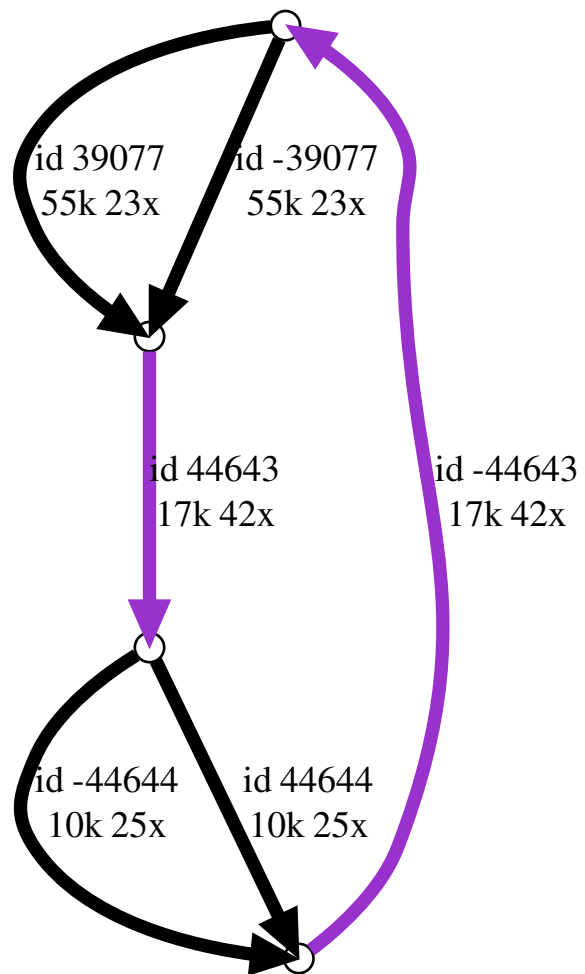

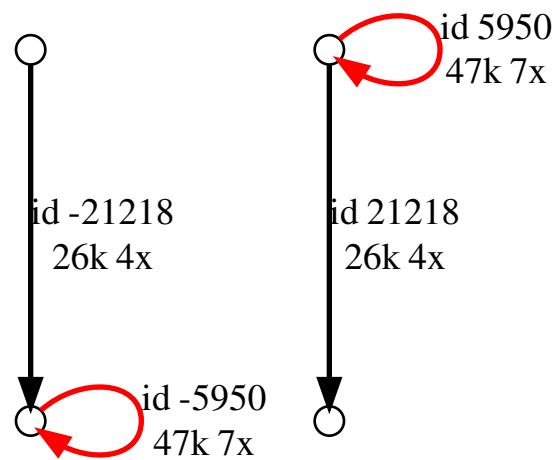

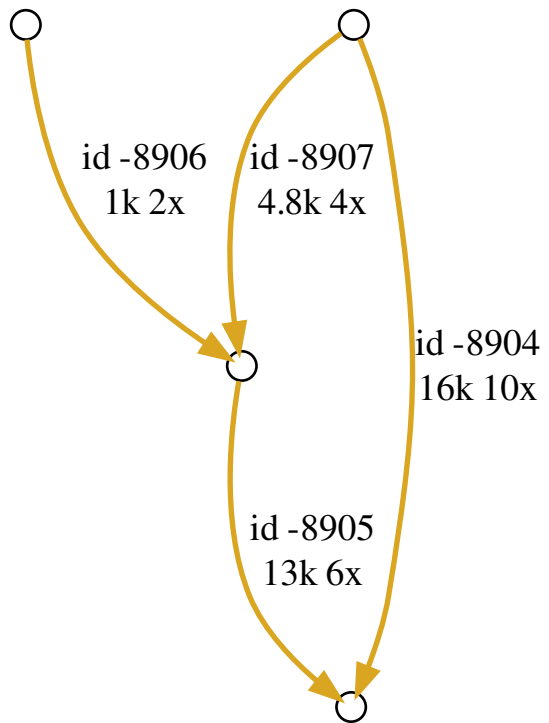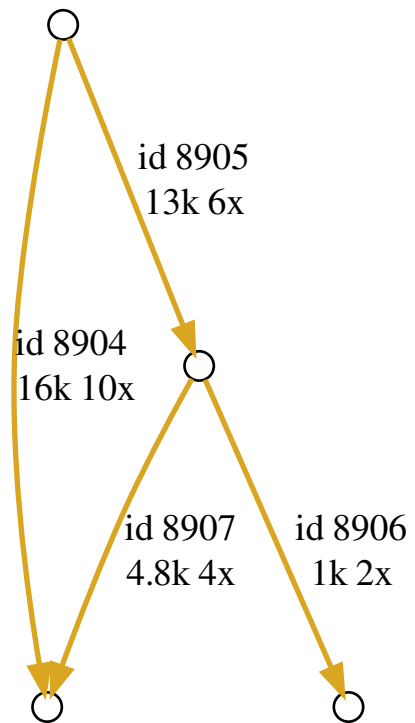

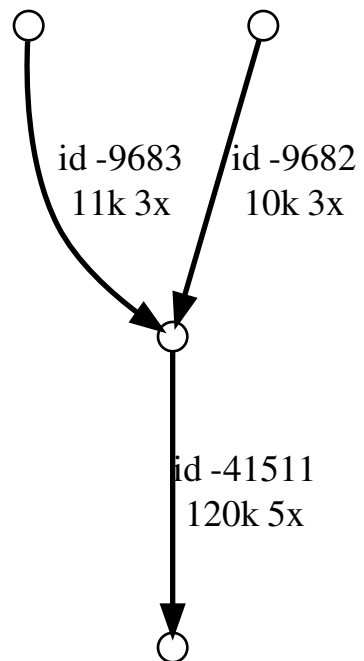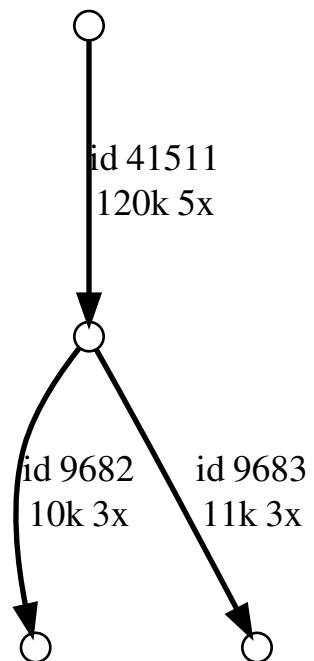

Supplement: Supplementary file 1 — Additional file 1 AGB vizualization of all viral components from SHEEP_GUT dataset. [file 13059_2021_2566_MOESM1_ESM.pdf]
