# Supplementary material for: viralFlye: assembling viruses and identifying their hosts from long-read metagenomics data
Source: Genome Biol. 2022 Feb 21;23:57. doi: 10.1186/s13059-021-02566-x (PMC8862349; doi:10.1186/s13059-021-02566-x)
Supplement: Supplementary file 4 — Additional file 4 Review history. [file 13059_2021_2566_MOESM4_ESM.docx]

Review History

**First round of review**

**Reviewer 1**

**Were you able to assess all statistics in the manuscript, including the appropriateness of statistical tests used?** There are no statistics in the manuscript.

**Were you able to directly test the methods?** No.

**Comments to author:**

Overall impressions

This paper by Antipov et al describes an alternative method to assemble virus genomes within long-read data and uses three publicly available data sets to compare the performance of the new assembler against current methods. Further, they perform host prediction on the newly identified viruses using a CRISPR approach and show an increase in the number of host matches that can be found.

The addition of a virus-specific module for metaflye is a welcome one for the research community and this paper has great potential; however from the tests that the authors provided, I'm still unsure of the performance gains provided by the viralFlye. Very few statistical testing. I detail these concerns below. Though I appreciate that the authors included the CRISPR analysis to show that better assembly leads to an increased number of virus-host predications, it is not very well fleshed out and feels like a last-minute thought, and most importantly does not give me significantly more confidence in the performance gains of viralflye. Overall, the paper lacks clarity in the results, and could use much more quantitative statements and statistics, and better introduction to the tools they use. I found that a lot of the really interesting parts of the paper are in fact in the methods, where most of the confusion I had while reading the result were resolved.


Major comments

1) Improve clarity: The results start with a description of the datasets (which can all be in the methods). Instead, I would start with a brief description of viralflye, and how it differs from your previous version. You do this clearly in the methods way below. Then, introduce your datasets, with an emphasis that each actually represent a different read sequencing technology (pacbio, nanopore, pacific bioscience). The fact that you tested multiple technologies in your benchmarking should be put forward much more, even in your conclusion. There are other issues, but these are in the line by line 'minor comments' section of this review. In addition, could you elaborate on how the long-read sequencing technology impacts viralflye's performance?
2) More assembler comparisons: It is hard to follow which study use which assembler; but most seem to have used metaflye. What about other assemblers or pipelines? E.g. Canu? It is very surprising that there is no 'quast-like' summary between viralflye and other assemblers!!
3) Table 1+2. I feel these could be a supplementary table, and for the main text, highlight all 3 datasets (the sheep dataset has no table!!!??), and the most important data, between viralflye and other assemblers.
4) The CRISPR analysis is lacking. The premise for omitting the human and marine datasets from the crispr analysis is wrong: just because these are viral metagenomes does not mean one cannot compare crisprs from it against public database of the hosts. Though it is less efficient than having a companion microbial metagenome (or using a bulk metagenome instead of a virome). Given that the main result here, for a single sample, is 15 vs 8 virus-host pairs detected, it falls short of being a convincing argument. I would rather the authors briefly mention this, and instead focus on establishing that viralflye perform better against other assemblers.

Minor comments:

PAGE1
L22. (abstract). 'it recovers many more viral genomes'. Can you more quantitative here?
L34-39. The inclusion of MAVs in the official ICTV taxonomy is still very little. Unless you have numbers to back up your statement, please remove. Alternatively, you could say that since there is a push from the ICTV to include whole-genome taxonomy from metagenomes, we better have good assembly tools. We are still far from MAVs being commonly accepted in ICTV.
L38. There is no 'universal viral classification system', as no universal virus gene exists, and virus taxonomic rules vary between virus groups. Please remove, and/or rephrase.
L40. 'MAVs are error-prone'. Can you explain more and provide references? You explain the phenomenon a little bit below, but this statement placed by itself feels off and unsubstantiated.
L41. ICTV instead of ICVT
L45-46: 'metagenome (virome)'. Please make sure to be clear here: a metagenome can be 'bulk' (ie, both microbial +virus), whereas virome is short for 'viral metagenome'. So here I think you want to add 'viral' in front of metagenome (virome).
L52-53: which sequencing technologies?

PAGE2
L8. There seems to be a reference missing here: #23, which is one of the studies you used.
L17. 'many more'. Please be specific
L20.'improve the accuracy'. Please be specific.
L61. Can you briefly explain in the text what viral complete does? (somewhere else you also use checkV…why?)
L62. What assembled was used?? This is part of one of the major comments; I recommend the authors to take the data, run it through multiple assemblers, and put all three samples in the same table of other figure if they wish (table 2 would be much better as a bar chart for example).

PAGE 3
L5. Please define 'multi-edge' in the main text and in the figure legend. Though you explain it in the methods, its biological meaning (e.g. viral strain) is super significant, and viralflye can 'see' them in the graphs; however in the current draft, it is a small aspect. The paper would be much more impactful and informative if you put this more forward, especially if biologists/virologists read you paper in trying to decide which assembler to use!
L38. 'assembled by 'metaflye'. Do you refer to the current metaflye? Or is viralflye separate? Or is viralflye an option in metaflye? You could clarify this in the beginning when you introduce the tool.
L46. Please briefly introduce 'circular disconnector'. At this stage the reader has no idea what it is and what it does.
L56. 'for polishing the long-read metaflye assemblies (an optional step in viral flye)'. I'm lost in knowing which tool does what. Please be clear.

PAGE4
Table 2. perhaps a bar chart would do better; and please provide full legends. A table legend should stand on their own.
L26. In addition to the similarity threshold, what there are length coverage threshold? Ie percent length of query against subject
L27. 'to be different. What does that mean? Do you mean there were no common MAVs between viralfly and ??.
L32. What is the meaning of 17/7/17?
L50-51. You now introduce checkV to measure genome completeness, but so far you have been reporting numbers from viral complete…please explain your reasoning.
L57. Is 2068 in parenthesis the number of linear contigs? Please re-phrase for clarity.
L58. Again, when you mention metaflye here, is it the 'old' version of the new version that contains viralflye???

PAGE 5
L34. There are many virus identification tools out there.which you could cite. In addition, how about just making viralfly assemble all the data WITHOUT a virus identication step in the middle, and leave viral identification to other tools after assembly?
L38. Period instead of comma after analysis.
L40. As mentioned above, this statement is incorrect. Once can totally predict host using criprs if you only have a virus metagenome.

PAGE6
L31. You mention that your significantly improved MAV reconstruction; however, the number of datasets are low (n=3), no statistical analysis was provided, and few quantitative mentions on the actual improvements of viralflye are provided. Please rephrase.
L32. 'previously described approaches'. Which ones? And how did viralflye achieve this?
L35. What limitations?
L43-45. Your strain argument here is very good, and is one of the biggest reasons why long-read are so important for viruses. I strongly recommend you talk more about this in your introduction. It's a very NB point!!
L56-60. This is the conclusion, not the results section. Otherwise, sum all these numbers in one sentence. This is too much results for the conclusion.

PAGE7
L28. How does viralflye deal with sequencing error? You do not mention this anywhere.

Page 8
L37. You select between 5kb-1mb. Viruses can be as small as 1.5kb (lots of circular ssDNA, foe which viralflye could be useful!!). Consider giving the choice as an option in the software…
L49-52. Again, perhaps would be good to give the freedom of choice here to go below 50%.
PAGE9.
L6-9. A figure of the viralflye pipeline would go a long way to better understand how it works.

General method: how where the reads processed? In what format were they? E.g. were these basecalled with the same software and version? These are critical differences that can infer from the nanopore data for example, and are not mentioned in the manuscript.

Note:
I could not test your software; I wish I could but when I tried to install it there were many dependency issues. Do you have a wrapper or docker/singularity/conda??

**Reviewer 2**

**Were you able to assess all statistics in the manuscript, including the appropriateness of statistical tests used?** There are no statistics in the manuscript.

**Were you able to directly test the methods?** No.

**Comments to author:**

This manuscript describes viralFlye, an adaptation of the popular metaFlye algorithm for long read metagenome assembly. ViralFlye is specialized in viral metagenome assembly using long read sequencing and is the first of its kind. The authors show that viralFlye assembles many more viral genomes than earlier publications on the same datasets. As such, it is a promising tool for the viral metagenomics community.

However, the software repository lacks a user manual; it only lists dependencies and describes the output, but there is no information on how to run the software. Even better would be to have some test data included, such that users can easily test if the installation is successful. Moreover, in order to allow for reproducibility, the authors should provide software versions and the exact command lines used for all steps to obtain the results presented in the manuscript.

I have some further concerns and questions per section:

Abstract: while the paper is generally very clear, the abstract needs some work to make it easier to read and more coherent.

Introduction:
The introduction is great on the metagenome assembly part, but the CRISPR spacer analysis for host-virus association comes out of the blue, this needs some further introduction. Moreover, this association is between phages and bacteria, and the manuscript should explain these specifics.

Results:
- It seems that the paper was written with the methods section before the results, and then sections were rearranged without further adaptation. This makes the results section hard to read. Both the results and the methods section require some rewriting to create a better flow. For example, it would be very helpful if the results section would start with a method overview, ideally with a workflow figure. Terms like ViralComplete, ViralDisconnector, DTR, TDR (typo?) can then be defined before use in the results part.
- In the results section it is not clear how assemblies are evaluated. This is described to some extent in the methods section, but I think it's important to have the evaluation criteria clearly described before discussing the results.
- One thing that remains unclear, even after reading the methods section, is how the assembly accuracy is verified? ViralFlye produces many additional contigs, but how do you assure that these are correct?

Methods:
- The methods section mentions that freebayes is used for short read polishing, but does not specify which aligner was used to perform the alignments.
- The component coverage computation uses edge coverage, how do you define and compute this?
- "many linear viruses have DTRs on both ends that often span 10 kb and more in length". I would replace "often span" by "can span". This is only true for larger viruses; for example, RNA viruses are generally only 10 kb in length in total.
- bottom of page 10: "with length varying from 5 kbp to 1 Mb for single contigs and to 500 Kbp for multi-edge viral components". Why is the threshold lower for multi-edge components? I would expect the opposite, since multi-edge components are likely a mix of multiple genomes, the total size can be larger?

**Authors Response**

We have now addressed all of the suggestions and revised the paper as requested. Below we provide a point-to-point response to the issues raised by reviewers. To simplify the work of reviewers, we have submitted both the revised manuscript and the black-and-blue version of this manuscript that shows all changes in blue.

**Reviewer #1:**

*Overall impressions*

This paper by Antipov et al describes an alternative method to assemble virus genomes within long-read data and uses three publicly available data sets to compare the performance of the new assembler against current methods. Further, they perform host prediction on the newly identified viruses using a CRISPR approach and show an increase in the number of host matches that can be found.

The addition of a virus-specific module for metaFlye is a welcome one for the research community and this paper has great potential; however from the tests that the authors provided, I'm still unsure of the performance gains provided by the viralFlye. Very few statistical testing. I detail these concerns below. Though I appreciate that the authors included the CRISPR analysis to show that better assembly leads to an increased number of virus-host predictions, it is not very well fleshed out and feels like a last-minute thought, and most importantly does not give me significantly more confidence in the performance gains of viralflye. Overall, the paper lacks clarity in the results, and could use much more quantitative statements and statistics, and better introduction to the tools they use. I found that a lot of the really interesting parts of the paper are in fact in the methods, where most of the confusion I had while reading the result were resolved.

*Major comments*

1)	Improve clarity: The results start with a description of the datasets (which can all be in the methods). Instead, I would start with a brief description of viralflye, and how it differs from your previous version. You do this clearly in the methods way below.

Thank you! To simplify the reviews, we have submitted both the revised manuscript and the black-and-blue version of this manuscript that shows all changes in blue.

We agree that a brief description of Methods before Results would make it easier to follow the paper. In the revised version, we have rearranged the text by placing a brief overview of Methods in the Results section.

A complete viral genome can be represented as an isolated contig (either linear or circular). However, if a viral genome is heterogeneous (represented by multiple closely-related strains or species), it may form multi-edge connected components in the metagenome assembly graph. In contrast to previous approaches that mainly focused on single contigs, the viralFlye pipeline analyzes both single viral contigs and multi-edge viral components. It includes the following steps described in details in the Methods section and illustrated in Figure 1:

1 Launching the modified metaFlye assembler (that we refer to as metaFlye v) aimed at viral genome sequencing.
2 Polishing the metaFlye assembly with short reads (optional step if short reads are available).

3 Identifying isolated linear and circular contigs in the assembly graph.

4 Identifying multi-edge connected components in the assembly graph (the ComponentTraverser module).

5 Detecting MAVs by verifying the identified linear and circular contigs and connected components as MAVs using the viralVerify tool [10] and checking them for completeness using the viralComplete tool [10].

6 Classifying viral genomes into linear and circular (the CircularDisconnector module).

7 Predicting virus-host association based on CRISPR analysis (optional step).

Then, introduce your datasets, with an emphasis that each actually represent a different read sequencing technology (pacbio, nanopore, pacific bioscience). The fact that you tested multiple technologies in your benchmarking should be put forward much more, even in your conclusion. There are other issues, but these are in the line by line 'minor comments' section of this review. In addition, could you elaborate on how the long-read sequencing technology impacts viralflye's performance?

We have now put more emphasis on various sequencing technologies, as suggested and extended our analysis by adding one more recently published dataset. We now provide expanded descriptions of how the datasets were originally generated in the “Datasets” subsection.

We benchmarked viralFlye using the following four datasets that cover various types of the currently available long read technologies.

WARWICK VIROME: Long error-prone reads from three marine water samples from English Channel, filtered using 0.22 mkm pore-size membrane, analyzed in [6]. DNA samples underwent whole genome amplification and were treated by S1 nuclease digestion to de-branch chimeric DNA formed during the rolling circle amplification process. MinION libraries were prepared using EXP-NBD104 and SQK-LSK109 kits and sequenced using MinION with a FLO-MIN106 flowcell. Datasets ERR6018294, ERR6018309, and ERR6018338 (referred to as WARWICK VIROME1, WARWICK VIROME2 and WARWICK VIROME3) contain 5.8, 6, and 7.3 Gb of reads respectively

2)	More assembler comparisons: It is hard to follow which study use which assembler; but most seem to have used metaflye. What about other assemblers or pipelines? E.g. Canu? It is very surprising that there is no 'quast-like' summary between viralflye and other assemblers!!

As we learned in our study of short-read plasmid (Antipov et al., *Genome Res.* 2019) and viral (Antipov et al. *Bioinformatics* 2020) assemblies, benchmarking specialized assemblers is challenging since there are no “gold standard” reference datasets. As a result, QUAST and metaQUAST have limited applicability in our analysis since these tools are most useful when the reference genomes are available.

We have now compared viralFlye with Raven and earlier version of metaFlye (without additional modifications introduced in this manuscript) as the reviewer suggested (tables 1-4). We note that it is a somewhat convoluted comparison since Raven and metaFlye are general purpose assemblers that do not include a specialized virus identification module. That is why previous long-read viral studies (Suzuki et al., 2019, Beaulaurier, Genome Res. 2020) introduced custom-built specialized pipelines for viral genome sequencing.

We thus used a pipeline that combines Raven (or metaFlye) with the same downstream analysis steps as in the viralFlye pipeline. We also note that these benchmarking results should be interpreted with caution.

We also launched Canu on all datasets but terminated it after running for more than 2 weeks without significant progress on all datasets except for the HiFi SHEEP_GUT dataset that took 6 days to assemble. This large running time is consistent with the previous reports of the inferior Canu performance on high-coverage metagenomic datasets (e.g. Kolmogorv et al, 2020, Bickhart et al., 2019).

We note that, in difference from all other long-read assemblers, Raven does not output the coverage of the assembled contigs and instead reports the number of reads that were used to generate each contig. The missing information about coverage (and a challenge of accurate read mapping to an assembly graph) makes it difficult to generate an apple-to-apple Raven benchmarking, e.g., the viralFlye pipeline uses a coverage cutoff to throw out the low-coverage contigs that often have misassemblies and increased number of single-base errors. We thus used the average read length for each dataset to approximate the coverage of Raven contigs using information about the read count. We acknowledge that this approximation becomes inaccurate when the read length is highly non-uniform.

We have added in the main text the following:

We have compared viralFlye with Raven [28] and the earlier version of metaFlye (i.e., the version metaFlye 2.8.3 that does not include additional modifications for viral assembly). We note that it is a somewhat convoluted comparison since Raven and metaFlye are general purpose assemblers that do not include a specialized virus identification module. We thus used pipelines (referred as Raven+viralTools and metaFlye+viralTools) that combine Raven and metaFlye with the same downstream analysis steps as in the viralFlye pipeline.

...

We note that viralFlye requires information about the contig coverage by reads. Since Raven does not report this information and only provides the read count readCount for each contig, we estimate the contig coverage in the Raven assembly using the average read length for each dataset (computed as *readCount* × *readLength*/*contigLength*). This approach may result in a biased estimate of the coverage since the read length for all analyzed datasets has a rather high variance.

3)	Table 1+2. I feel these could be a supplementary table, and for the main text, highlight all 3 datasets (the sheep dataset has no table!!!??), and the most important data, between viralflye and other assemblers.

There was no Table for the SHEEP_GUT dataset since, in contrast to the two other datasets, there was no previously published specialized viral pipeline to compare the results. In the revised version, we are using the Raven-based and metaFlye-based pipeline for benchmarking on the sheep dataset. The updated Tables 1-4 now report the information about all four datasets (in the same style). We felt that combining the four tables into a single large Table would make it somewhat difficult to read.

4)	The CRISPR analysis is lacking. The premise for omitting the human and marine datasets from the crispr analysis is wrong: just because these are viral metagenomes does not mean one cannot compare crisprs from it against public database of the hosts. Though it is less efficient than having a companion microbial metagenome (or using a bulk metagenome instead of a virome). Given that the main result here, for a single sample, is 15 vs 8 virus-host pairs detected, it falls short of being a convincing argument. I would rather the authors briefly mention this, and instead focus on establishing that viralflye perform better against other assemblers.

As the reviewer suggested, we have now added a Supplement tables S2-S4 that describe the comparison with public CRISPR database (for HUMAN_GUT, ALOHA_VIROME and WARWICK_VIROME datasets) in the “known host-novel virus” mode that works well for both short and long reads. However, the power of long reads is best illustrated by the “novel host-novel virus” mode that requires a well-assembled bacterial metagenome from the same sample and can only be done in the case of the SHEEP_GUT sample. Since it is a new mode in the virus-host association studies, we would prefer to keep it in the main text.

With respect to a seemingly small increase in the number of the identified virus-host pairs between MAVs identified by both long and short reads, we note that, as Table 5 illustrates, the overlap between short-read MAVs and long-read MAVs is surprisingly small. We were puzzled by this result but a recent study of the same sheep dataset in Pevzner’s lab shed some light on the reason for such a small overlap (Bickhart et al., *Nature Biotechnology*, in press). It turned out that the coverages of various genomes are highly non-proportional between short and long reads. As a result, some viruses may be well-covered by short reads and poorly-covered by long reads (and vice versa) and thus be missed in long-read assemblies.

We thus believe that the most important result in Table 5 is given in its first (rather than third) row - it illustrates that the “novel host-novel virus” mode (enabled by long reads) is an important complement to the popular “known host-novel virus” mode since the latter mode misses many virus-host associations.

The text

CRISPR-based host prediction

Unfortunately, the MARINE VIROME and the HUMAN GUT datasets are not well-suited for CRISPR analysis, Indeed, since the MARINE VIROME dataset was size-selected for viruses, the potential hosts in this dataset are filtered out. Since the HUMAN GUT dataset has a rather low coverage by short reads (0.7-3.2 Gb per sample for the HUMAN GUT dataset as compared to 159.3 Gb bases per sample for the SHEEP GUT dataset), we did not identify complete viruses present in both short- and long-read assemblies. We thus limited analysis of virus-host associations to the SHEEP GUT dataset (both short- and long-read metagenomic assemblies)

was rearranged as follows:

CRISPR-based host prediction

Identification of known CRISPR spacers in MAVs is a powerful approach for predicting potential known hosts for novel viruses from metagenomic assemblies [1, 31]. However, this approach is limited by the CRISPR arrays available in the existing (rather incomplete) CRISPR databases. Here we demonstrate that the novel CRISPR arrays identified in a newly assembled metagenomic sample provide additional and more accurate information for predicting potential novel hosts for novel viruses. We focused on this database-free “novel host-novel virus” mode, and compared it with the traditional database-based “known host-novel virus” approach. For the SHEEP GUT dataset, where both long and short reads (with the high coverage) were available, we studied intra-sample virus-host associations for both types of data, and compared them with the results obtained using the CRISPRCasdb database (see Methods for details).

Since the ALOHA_VIROME and WARWICK_VIROME datasets were size-selected for viruses, the potential hosts in this dataset are filtered out. Since the HUMAN_GUT dataset has a rather low coverage by short reads (0.7-3.2 Gb per sample for the HUMAN_GUT dataset as compared to 159.3 Gb bases per sample for the SHEEP GUT dataset), we did not identify complete viruses present in both short- and longread assemblies. Thus, since the ALOHA_VIROME, WARWICK_VIROME and the HUMAN_GUT datasets are not well-suited for CRISPR analysis, we limited the analysis of corresponding MAVs by the traditional “known host-novel virus” approach ([Supplemental tables 2-4](https://docs.google.com/spreadsheets/d/1LBkRTzhTs3OrCrQOQljhwfcXfDwfXYwUVDBQM69gWdQ/edit?usp=sharing)).

*Minor comments:*

PAGE1

L22. (abstract). 'it recovers many more viral genomes'. Can you be more quantitative here?

We tried to rephrase this non-quantitative statement. However, it is difficult to do it in the space-constrained Abstract since different datasets are characterized by varying rates of increases. We thus reworded this sentence

We describe the viralFlye tool for identifying metagenome-assembled viruses in long-read assemblies and show that it recovers many more viral genomes than the previous studies.

as follows:

We describe the viralFlye tool for identification and analysis of metagenome-assembled viruses (MAVs) in long-read assemblies, show that it significantly improves viral assemblies, and demonstrate that long reads result in a much larger array of predicted virus-host association as compared to short-read assemblies.

L34-39. The inclusion of MAVs in the official ICTV taxonomy is still very little. Unless you have numbers to back up your statement, please remove. Alternatively, you could say that since there is a push from the ICTV to include whole-genome taxonomy from metagenomes, we better have good assembly tools. We are still far from MAVs being commonly accepted in ICTV.

We have revised this paragraph

Since metagenomic sequencing has greatly expanded the set of known viral genomes (Paez-Espino et al., 2016; Roux et al., 2016, Edgar, 2020, Yutin, 2021), the International Committee on Taxonomy of Viruses (ICTV) decided that novel viruses can now be described based solely on the metagenomic sequence data (Simmonds et al., 2017). As a result, the universal viral classification system now incorporates many *metagenome-assembled viruses* (*MAVs*). However, since MAVs are error-prone, this ICVT decision emphasized the need for novel bioinformatics tools to accurately identify, verify, analyze, and classify novel viruses.

as follows.

Various metagenomic studies have greatly expanded the set of known viral genomes (Paez-Espino et al., 2016; Roux et al., 2016, Edgar, 2020, Yutin, 2021, Benler 2021, Nayfach et al., 2021, Michniewski et al., 2021) and have raised the challenge of inferring *metagenome-assembled viruses* (*MAVs*). Since the International Committee on Taxonomy of Viruses has proposed to include MAVs into viral taxonomy studies (Simmonds et al., 2017), there is a need for novel bioinformatics tools to accurately assemble, identify, verify, analyze, and classify MAVs.

L38. There is no 'universal viral classification system', as no universal virus gene exists, and virus taxonomic rules vary between virus groups. Please remove, and/or rephrase.

We have removed it as the reviewer proposed.

L40. 'MAVs are error-prone'. Can you explain more and provide references? You explain the phenomenon a little bit below, but this statement placed by itself feels off and unsubstantiated.

We have removed this statement from the first paragraph as it is explained later in the manuscript.

L41. ICTV instead of ICVT

Revised as suggested.

L45-46: 'metagenome (virome)'. Please make sure to be clear here: a metagenome can be 'bulk' (ie, both microbial +virus), whereas virome is short for 'viral metagenome'. So here I think you want to add 'viral' in front of the metagenome (virome).

Revised as suggested.

L52-53: which sequencing technologies?

These viruses were sequenced by a combination of Illumina/454/PacBio (first one) and Sanger/PCR/cloning for gap closing (second one). Since it is not the focus of our paper, we shortened this sentence

For example, complete sequencing of the giant viruses (Raoult et al, 2004, Philippe et al, 2013) has been a challenging task that required a combination of multiple sequencing technologies.

as follows:

For example, complete sequencing of giant viruses has been a challenging task (Raoult et al, 2004, Philippe et al, 2013).

PAGE2

L8. There seems to be a reference missing here: #23, which is one of the studies you used.

Thank you, fixed in text

L17. 'many more'. Please be specific

Thank you, rephrased

recovers up to 2.25 times more complete

L20.'improve the accuracy'. Please be specific.

We revised this sentence

We also show that long reads improve the accuracy of virus-host association predictions based on matching of CRISPR-Cas sites.

as follows:

We also show that long reads improve the accuracy of the virus-host association predictions (i.e., enable the host identification at a deeper taxonomic level) based on matching of the CRISPR-Cas sites.

L61. Can you briefly explain in the text what viral complete does? (somewhere else you also use checkV…why?)

The viralComplete step in the viralFlye pipeline implements a similarity-based check of the completeness of a putative viral sequence. We hope that after moving the description of the pipeline to the beginning of Results, the role of the viralComplete step is clear. checkV was used as an orthogonal benchmarking approach. Tables 1-4 illustrate that checkV and viralComplete are producing consistent results, with checkV being slightly more conservative in its predictions.

L62. What assembler was used?? This is part of one of the major comments; I recommend the authors to take the data, run it through multiple assemblers, and put all three samples in the same table of other figure if they wish (table 2 would be much better as a bar chart for example).

It’s difficult to compare very different pipelines and approaches since analysis in previous papers included significant manual work and in-house scripts. For example, the HUMAN_GUT dataset was initially assembled with modified FALCON, complemented by unpublished in-house scripts and manual analysis. The assembly-free pipeline developed for initial analysis of the ALOHA_VIROME dataset is only applicable to linear viruses. We thus provided comparison with results from original papers in the original submission and have now complemented it by comparison with other general-purpose assemblers (Tables 1-4).

PAGE 3

L5. Please define 'multi-edge' in the main text and in the figure legend. Though you explain it in the methods, its biological meaning (e.g. viral strain) is super significant, and viralflye can 'see' them in the graphs; however in the current draft, it is a small aspect. The paper would be much more impactful and informative if you put this more forward, especially if biologists/virologists read your paper in trying to decide which assembler to use!

Thanks, we have defined multi-edge components and provided a biological interpretation in the beginning of the Results section as follows:

A complete viral genome can be represented as an isolated contig (either linear or circular). However, if a viral genome is heterogeneous (represented by multiple closely-related strains or species), it may form multi-edge connected components in the metagenome assembly graph. In contrast to previous approaches that mainly focused on single contigs, the viralFlye pipeline analyzes both single viral contigs and multi-edge viral components.

L38. 'assembled by 'metaflye'. Do you refer to the current metaflye? Or is viralflye separate? Or is viralflye an option in metaflye? You could clarify this in the beginning when you introduce the tool.

We apologize for the confusion. In the revised text we denote the modified version of metaFlye as metaFlye_v (and clarified this in the beginning of the Results section). The introduced algorithmic changes are outlined in the Results subsection “Modifying metaFlye for viral assembly”.

L46. Please briefly introduce the 'circular disconnector'. At this stage the reader has no idea what it is and what it does.

Thanks, we reordered the text to introduce parts of the viralFlye pipeline first.

L56. 'for polishing the long-read metaflye assemblies (an optional step in viral flye)'. I'm lost in knowing which tool does what. Please be clear.

We have added a new Figure illustrating all steps of viralFlye and outlined its steps in the beginning of the Results section.

**Figure 1. Graphical representation of the viralFlye pipeline.**

PAGE4

Table 2. perhaps a bar chart would do better; and please provide full legends. A table legend should stand on their own.

Thank you, we’ve updated the table legend.

L26. In addition to the similarity threshold, what there are length coverage threshold? Ie percent length of query against subject

We did not use the “length coverage threshold” since the CD-HIT-EST tool uses the global sequence identity by default. We’ve added all the settings and software versions in the new Supplementary file 2.

Supplementary file 2 contains information about all default settings and versions used for each software tool.

Metaflye_v : metaFlye build 2.9-b1774, (git revision af269a3470db78c9c95934e1a5aa5ce218640804 ) with --scaffold option

For the viralTools pipeline we used the latest versions of the following software tools available in conda:

Minimap2: 2.22-r1101

Prodigal: V2.6.3

viralVerify 1.1

Samtools& bcftools 1.13

Seqtk 1.3-r106

Minced 0.4.2

Freebayes v0.9.21

CD-HIT-EST v.4.8.1, similarity threshold 0.95

CheckV v0.7.0

Blastn, v. 2.6.0+, max evalue 1E-5, min identity 0.9, task = blastn-short

All these tools were run with their default settings unless otherwise specified

L27. 'to be different. What does that mean? Do you mean there were no common MAVs between viralfly and ??.

Indeed, no MAVs with similarity>0.95, now clarified in text

All MAVs identified by viralFlye turned out to be different

No MAVs identified by viralFlye had similarity exceeding > 0.95, implying that they represent different viruses.

L32. What is the meaning of 17/7/17?

These are numbers for 3 datasets, rephrased. Numbers changed after further software development. The corresponding paragraph is rewritten as follows:

CircularDisconnector labeled 17/7/17 circular contigs in MARINE VIROME1, MARINE VIROME2, and MARINE VIROME3 datasets

CircularDisconnector labeled 28, 4 and 26 circular contigs in ALOHA_VIROME1, ALOHA_VIROME2, and ALOHA_VIROME3 datasets as falsely circularized linear viral contigs.

L50-51. You now introduce checkV to measure genome completeness, but so far you have been reporting numbers from viral complete…please explain your reasoning.

We check whether the additional contigs represent complete viruses using checkV (which is not a part of the pipeline but used for benchmarking only). Tables 1-4 now report the statistics from both viralComplete and CheckV.

L57. Is 2068 in parenthesis the number of linear contigs? Please re-phrase for clarity.

Yes, it is the number of linear conigs. Also, with all additional modification and filtering by length and coverage, the number of isolated circular and linear contigs decreased, and now the corresponding paragraph is rewritten as follows:

We note that there are 153 high-coverage circular contigs shorter than 1Mb in the metaFlye_vv assembly

…

On the other hand, metaFlye_v generated 158 non-characterized high-coverage linear contigs in the SHEEP_GUT dataset.

L58. Again, when you mention metaflye here, is it the 'old' version of the new version that contains viralflye???

We have now replaced all “modified metaflye” occurrences with “metaFlye_v”.

PAGE 5

L34. There are many virus identification tools out there.which you could cite. In addition, how about just making viralfly assemble all the data WITHOUT a virus identication step in the middle, and leave viral identification to other tools after assembly?

Thanks, we cited a few viral identification tools - . An experienced user should be able to alter the viralFlye pipeline by substituting viralVerify with an alternative viral identification tool.

L38. Period instead of comma after analysis.

Thank you, fixed

L40. As mentioned above, this statement is incorrect. Once can totally predict host using criprs if you only have a virus metagenome.

Here we refer to the impossibility to find potential hosts in the *same* sample. One can indeed use CRISPR databases for host prediction but we want to extend this popular approach by finding potential hosts (even unknown ones) in the *same* sample.

PAGE6

L31. You mention that your significantly improved MAV reconstruction; however, the number of datasets are low (n=3), no statistical analysis was provided, and few quantitative mentions on the actual improvements of viralflye are provided. Please rephrase.

We removed “significantly” from the text.

Nevertheless, we have shown that viralFlye significantly improves reconstruction and analysis of MAVs as compared to the previously described approaches to MAV identification in long-read assemblies.

Nevertheless, we have shown that viralFlye improves reconstruction and analysis of MAVs as compared to the previously described approaches [15, 27, 14] to MAV identification in long-read assemblies.

L32. 'previously described approaches'. Which ones? And how did viralflye achieve this?

Added citations for the following papers:

Warwick-Dugdale, J., Solonenko, N., Moore, K., Chittick, L., Gregory, A.C., Allen, M.J., Sullivan, M.B.,Temperton, B.: Long-read viral metagenomics captures abundant and microdiverse viral populations and their niche-defining genomic islands. PeerJ7, 6800 (2019)

Suzuki, Y., Nishijima, S., Furuta, Y., Yoshimura, J., Suda, W., Oshima, K., Hattori, M., Morishita, S.:Long-read metagenomic exploration of extrachromosomal mobile genetic elements in the human gut. Microbiome 7(1), 1–16 (2019)

Beaulaurier, J., Luo, E., Eppley, J.M., Uyl, P.D., Dai, X., Burger, A., Turner, D.J., Pendelton, M., Juul, S.,Harrington, E., DeLong, E.F.: Assembly-free single-molecule sequencing recovers complete virus genomes from natural microbial communities. Genome Research 30(3), 437–446 (2020)

L35. What limitations?

Rephrased in the revised manuscript as follows:

We showed that the previously developed custom pipelines for MAV generation have various limitations, leading to both missing and incorrectly assembled MAVs

We showed that the previously developed custom pipelines for MAV generation may lead to both missing and incorrectly assembled MAVs.

L43-45. Your strain argument here is very good, and is one of the biggest reasons why long-read are so important for viruses. I strongly recommend you talk more about this in your introduction. It's a very NB point!!

Thank you - we have added the following text to the Introduction:

Interestingly, viralFlye also revealed viruses that represent a population of multiple viral strains (species) that have structural variations or highly diverged regions. Although analysis of such populations may provide insights into how multi-strain viral communities are organized and how they evolve, viruses forming such populations typically remain below the radar of short-read studies.

L56-60. This is the conclusion, not the results section. Otherwise, sum all these numbers in one sentence. This is too much results for the conclusion.

Thank you, we removed the corresponding fragment.

30, 658 and 102 circular contigs in the HUMAN GUT, MARINE VIROME, and SHEEP GUT datasets, respectively. It also revealed 1454, 5139 and 192 uncharacterized isolated linear contigs in the HUMAN GUT, MARINE VIROME, and SHEEP GUT datasets, respectively.

PAGE7

L28. How does viralflye deal with sequencing error? You do not mention this anywhere.

Wrt sequencing errors, viralFlye benefits from the polishing module in metaFlye and complements it with a procedure for splitting chimeric reads that may span multiple linear viruses (described in Methods)

Page 8

L37. You select between 5kb-1mb. Viruses can be as small as 1.5kb (lots of circular ssDNA, for which viralflye could be useful!!). Consider giving the choice as an option in the software…

Thank you, we have now explicitly specified that a user can change the default parameters.

viralFlye extracts well-covered circular and linear isolated contigs from the GFA assembly files with length varying between 5 kb and 1 Mb

viralFlye extracts high-coverage circular and linear isolated contigs from the GFA assembly files with the lengths varying between *minLength* (default value 5 kb) and *maxLength* (default value 1 Mb). A user can change these default values, e.g., to analyze short circular ssDNA viruses, it would be useful to decrease the *minLength* parameter to 1.5 kb.

L49-52. Again, perhaps would be good to give the freedom of choice here to go below 50%.

We added the suggested option. The text has been revised as follows:

We have reduced the completeness threshold to 50%

Since metagenomic samples likely contain many viruses without close database references, we have reduced the completeness threshold to 50% and provided an option to change it.

PAGE9.

L6-9. A figure of the viralflye pipeline would go a long way to better understand how it works.

Thank you, we added this Figure to the Results section.

General method: how were the reads processed? In what format were they? E.g. were these basecalled with the same software and version? These are critical differences that can infer from the nanopore data for example, and are not mentioned in the manuscript.

Thank you, added the details. We used data from SRA, so they were already basecalled. We have clarified it in the Results section:

All datasets were downloaded from SRA in the fastq format.

Note:

I could not test your software; I wish I could but when I tried to install it there were many dependency issues. Do you have a wrapper or docker/singularity/conda??

Thank you, we have now improved the usability of viralFlye and provided the detailed documentation of the pipeline. As requested, it is now wrapped in a conda environment.

**Reviewer #2:**

This manuscript describes viralFlye, an adaptation of the popular metaFlye algorithm for long read metagenome assembly. ViralFlye is specialized in viral metagenome assembly using long read sequencing and is the first of its kind. The authors show that viralFlye assembles many more viral genomes than earlier publications on the same datasets. As such, it is a promising tool for the viral metagenomics community.

However, the software repository lacks a user manual; it only lists dependencies and describes the output, but there is no information on how to run the software. Even better would be to have some test data included, such that users can easily test if the installation is successful. Moreover, in order to allow for reproducibility, the authors should provide software versions and the exact command lines used for all steps to obtain the results presented in the manuscript.

Thank you! To simplify the reviews, we have submitted both the revised manuscript and the black-and-blue version of this manuscript that shows all changes in blue.

Since it was a submission to a Special Issue, we rushed and did not have time to document the viralFlye software. We have now updated the viralFlye description in Supplementary File 2 (software versions) and the manual.

I have some further concerns and questions per section:

Abstract: while the paper is generally very clear, the abstract needs some work to make it easier to read and more coherent.

The revised abstract is below. Since the journal has strict word limits (100 words) for abstracts of this type of manuscript, we tried to be concise and were not able to include all the details.

Although the long-read metagenomics improves the contiguity of the assembled viral genomes as compared to short-read methods, assembling complex viral communities remains an open problem. We describe the viralFlye tool for identification and analysis of metagenome-assembled viruses (MAVs) in long-read assemblies, show that it significantly improves viral assemblies, and demonstrate that long reads result in a much larger array of predicted virus-host association as compared to short-read assemblies.

Introduction:

The introduction is great on the metagenome assembly part, but the CRISPR spacer analysis for host-virus association comes out of the blue; this needs some further introduction. Moreover, this association is between phages and bacteria, and the manuscript should explain these specifics.

The very idea of the CRISPR analysis here was to improve the host prediction for novel MAVs based on the companion microbial metagenome. It is not directly related to MAV assembly. However, the power of long reads is best illustrated by the “novel host-novel virus” mode that requires a well-assembled bacterial metagenome from the same sample and can only be done in the case of the SHEEP_GUT sample. Table 3 illustrates that the “novel host-novel virus” mode (enabled by long reads) is an important complement to the popular “known host-novel virus” mode since the latter mode misses many virus-host associations.

Results:

- It seems that the paper was written with the methods section before the results, and then sections were rearranged without further adaptation. This makes the results section hard to read. Both the results and the methods section require some rewriting to create a better flow. For example, it would be very helpful if the results section would start with a method overview, ideally with a workflow figure. Terms like ViralComplete, ViralDisconnector, DTR, TDR (typo?) can then be defined before use in the results part.

We have now added a brief description of methods and a Figure illustrating the viralFlye pipeline at the beginning of Results.

- In the results section it is not clear how assemblies are evaluated. This is described to some extent in the methods section, but I think it's important to have the evaluation criteria clearly described before discussing the results.

We have added a brief description of evaluations at the beginning of Results, subsection “Benchmarking”. The subsection now now reads:

We have compared viralFlye with Raven [28] and the earlier version of metaFlye (i.e., the version metaFlye 2.8.3 that does not include additional modifications for viral assembly). We note that it is a somewhat convoluted comparison since Raven and metaFlye are general purpose assemblers that do not include a specialized virus identification module. We thus used pipelines (referred as Raven+viralTools and metaFlye+viralTools) that combine Raven and metaFlye with the same downstream analysis steps as in the viralFlye pipeline.

We evaluated the assemblies from all pipeline configurations in terms of the number of reconstructed viral genomes, identified by viralComplete. In addition, used the checkV tool [29] as an orthogonal validation approach. As we show below, viralFlye recovered the largest number of viral genomes for nearly all analyzed datasets, and had the lowest rate of chimeric misassemblies.

- One thing that remains unclear, even after reading the methods section, is how the assembly accuracy is verified? ViralFlye produces many additional contigs, but how do you assure that these are correct?

A standard QUAST-like evaluation was not possible for the analyzed metagenomic datasets, as high-quality reference genomes were not available (and we are not aware of any other real metagenomic datasets with known references). Instead, we checked whether the additional contigs represent complete viruses using checkV (which is not a part of the pipeline and used as an orthogonal evaluation method). In the Benchmarking section we have added the following:

In addition, we used the checkV tool [29] as an orthogonal validation approach.

In addition to the completeness statistics, we evaluated the rate of chimeric misassemblies using the likely-complete viral genomes from the ALOHA_VIROME dataset as follows:

We further used the assemblies from the original manuscript [27] to estimate the rate of chimeric misassemblies. We aligned the query contigs (in metaFlye, metaFlye v, and Raven assemblies)against the original (reference) contigs. All reference contigs contain direct terminal repeats (DTRs), therefore are likely to represent complete viruses. A query contig that is collinear to a concatenation of two different reference contigs is then classified as potentially chimeric. The metaFlye pipeline identified 81 potentially chimeric contigs, while viralFlye identified only 6, suggesting that the specialized viral assembler is less prone to misassemblies. The Raven pipeline identified 4 potentially chimeric contigs, but assembled an order of magnitude fewer viral genomes

Methods:

- The methods section mentions that freebayes is used for short read polishing, but does not specify which aligner was used to perform the alignments.

Revised as follows:
viralFlye uses the freebayes tool [30] followed by the bcftools consensus tool [31] for polishing.

viralFlye uses the freebayes tool [35] (with the bwa mem aligner [36]) followed by the bcftools consensus tool [37] for polishing.

- The component coverage computation uses edge coverage, how do you define and compute this?

For metaFlye and metaFlye_v we used the edge coverage reported by those tools. In the revised version of the manuscript we also included Raven assemblies in our benchmarks. Since Raven outputs read counts for each edge instead of coverage, we estimated the coverage as follows:

Since Raven does not report this information and only provides the read count *readCount* for each contig, we estimate the contig coverage in the Raven assembly using the average read length for each dataset (computed as *readCount* * *readLength* / *contigLength*). This approach may result in a biased estimate of the coverage since the read length for all analyzed datasets has a rather high variance

- "many linear viruses have DTRs on both ends that often span 10 kb and more in length". I would replace "often span" by "can span". This is only true for larger viruses; for example, RNA viruses are generally only 10 kb in length in total.

Revised as suggested

- bottom of page 10: "with length varying from 5 kbp to 1 Mb for single contigs and to 500 Kbp for multi-edge viral components". Why is the threshold lower for multi-edge components? I would expect the opposite, since multi-edge components are likely a mix of multiple genomes, the total size can be larger?

We assume that a multi-edge component is either a single viral genome that metaFlye failed to assemble in a single contig (because of internal repeats) or a group of similar viruses (strains) that share long highly-similar genomic regions. As suggested, in the revised version we have the same threshold (1 Mb) for both contigs and viral multi-edge components.

**Second round of review**

**Reviewer 1**

I commend the authors for the quality and depth of their revisions. I'm happy with the changes and recommend publication of this work.

**Reviewer 2**

Thank you for the clarifications, the revised manuscript reads very nicely. My only remaining comment is that tables 1-4 are a little hard to read, it would help if e.g. additional spacing or coloring was used to distinguish between the different methods.
